# Vasodilator Myocardial Perfusion Cardiac Magnetic Resonance Imaging Is Superior to Dobutamine Stress Echocardiography in the Detection of Relevant Coronary Artery Stenosis: A Systematic Review and Meta-Analysis on Their Diagnostic Accuracy

**DOI:** 10.3389/fcvm.2021.630846

**Published:** 2021-03-12

**Authors:** Sebastian M. Haberkorn, Sandra I. Haberkorn, Florian Bönner, Malte Kelm, Gareth Hopkin, Steffen E. Petersen

**Affiliations:** ^1^Department of Cardiology, Pneumology and Angiology, University Hospital Düsseldorf, Düsseldorf, Germany; ^2^Department of Health Policy, London School of Economics and Political Science, London, United Kingdom; ^3^William Harvey Research Institute, Queen Mary University of London, London, United Kingdom; ^4^Barts Heart Center, St. Bartholomew's Hospital, Barts Health NHS (National Health Service) Trust, London, United Kingdom

**Keywords:** meta-analysis, systematic (literature) review, dobutamine stress echocardiography, diagnostic test accuracy, cardiac imaging, coronary artery disease, cardiac MR, myocardial perfusion MR

## Abstract

**Objectives:** Guideline recommendations for patients with either a high or a low risk of obstructive coronary artery disease (CAD) are clear. However, the evidence for initial risk stratification in patients with an intermediate risk of CAD is still unclear, despite the availability of multiple non-invasive assessment strategies. The aim of this study was to synthesize the evidence for this population to provide more informed recommendations.

**Background:** A meta-analysis was performed to systematically assess the diagnostic accuracy of vasodilator myocardial perfusion cardiovascular magnetic resonance imaging (pCMR) and dobutamine stress echocardiography (DSE) for the detection of relevant CAD. In contrast to previous work, this meta-analysis follows rigorous selection criteria in regards to the risk stratification and a narrowly prespecified definition of their invasive reference tests, resulting in unprecedentedly informative results for this reference group.

**Data Collection and Analysis:** From the 5,634 studies identified, 1,306 relevant articles were selected after title screening and further abstract screening left 865 studies for full-text review. Of these, 47 studies fulfilled all inclusion criteria resulting in a total sample size of 4,742 patients.

**Results:** pCMR studies showed a superior sensitivity [0.88 (95% confidence interval (CI): 0.85–0.90) vs. 0.72 (95% CI: 0.61–0.81)], diagnostic odds ratio (DOR) [38 (95% CI: 29–49) vs. 20 (95% CI: 9–46)] and an augmented post-test probability [negative likelihood ratio (LR) of 0.14 (95% CI: 0.12–0.18) vs. 0.31 (95% CI: 0.21, 0.46)] as compared to DSE. Specificity was statistically indifferent [0.84 (95% CI: 0.81–0.87) vs. 0.89 (95% CI: 0.83–0.93)].

**Conclusion:** The results of this systematic review and meta-analysis suggest that pCMR has a superior diagnostic test accuracy for relevant CAD compared to DSE. In patients with intermediate risk of CAD only pCMR can reliably rule out relevant stenosis. In this risk cohort, pCMR can be offered for initial risk stratification and guidance of further invasive treatment as it also rules in relevant CAD.

## Key Points

- *Question:* Which imaging modality for initial risk stratification of patients with an intermediate pre-test probability of CAD is superior?
- *Findings:* In this systematic review and meta-analysis the diagnostic accuracy of pCMR and DSE was systematically assessed in 47 studies reporting data from 4,742 patients. The findings suggest that pCMR has a superior test accuracy compared to DSE in the detection of relevant CAD (sensitivity 0.88 vs. 0.72, specificity 0.84 vs. 0.89).
- *Meaning:* Despite the widespread use of DSE, the evidence at hand favors pCMR in the risk stratification of patients with an intermediate risk of CAD.

## Introduction

Myocardial ischemia in the form of relevant coronary artery stenosis is strongly associated with adverse outcomes, such as myocardial infarctions (MIs) and death (1). An early and accurate identification of myocardial ischemia has consequently been highlighted as a priority in current international guidelines (2, 3). Conventional coronary angiography (CCA) or a fractional flow reserve (FFR)-based assessment is the reference standard for diagnosis of CAD in patients with a high pre-test probability. An non-invasive assessment with multi-detector CT-angiography (MDCT) is the preferred approach in patients with a low pre-test probability of CAD (3).

However, in the large number of patients with an intermediate pre-test probability, guidance is underdeveloped on which of the different non-invasive imaging modalities is to prefer and clear recommendations are not yet available (2, 3). Myocardial perfusion cardiovascular magnetic resonance imaging (pCMR), dobutamine stress echocardiography (DSE), or alternative techniques can be performed equivalently for a non-invasive functional assessment of myocardial ischemia in this risk cohort (4). Therefore, there is a strong need to identify the best diagnostic alternative for these patients.

In this systematic review and meta-analysis, pCMR and DSE have been selected because they are the only imaging modalities without radiation and are frequently operated by cardiologists alone. Moreover, a diagnostic superiority over Single Photon Emission Computed Tomography (SPECT) in the detection of relevant coronary stenosis, for instance, has already been shown in several meta-analyses (4, 5), and trials, such as the CE-MARC, MR-IMPACT I, and II (6, 7). Most recently, the MR-INFORM trial even suggested that pCMR is “*non-inferior to FFR with respect to major adverse cardiac events*” in stable patients with high risk of CAD, underlining its significance in the non-invasive assessment of CAD (8).

Several systematic reviews and meta-analyses have been published on the diagnostic accuracy of various imaging approaches (4, 5, 9). However, the ability of these meta-analyses to support clinical decission making is potentially limited due to considerable heterogeneity between their included studies. This heterogeneity is due to broad eligibility criteria, varying reference tests and comparators, and vague definitions of “*significant coronary artery stenosis*” (5). Most importantly though, heterogeneity is due to individual patient risk for CAD in the included study cohorts, such as age, sex, and different risk factors. Studies with verification bias and studies from unsystematic literature searches are included in some of these analyses, which influences their applicability. Finally, previous meta-analyses have not employed systematic search methods, resulting in an incomplete or invalid identification of the available evidence. Each of these limitations of existing evidence reduces their ability to make recommendations for specific populations in the context of guideline development and as such there are still evidence gaps in the literature.

To address these existing evidence gaps, we performed a systematic review and meta-analysis with rigorous eligibility criteria on risk stratification and reference procedures, ascertaining the diagnosis of hemodynamically significant CAD. We focused on pCMR and DSE with the aim of providing resilient recommendations for the large number of patients with an intermediate risk of CAD.

## Methods

### Registration

This systematic review and meta-analysis was prospectively registered in PROSPERO under the registration number CRD42018105535. Reporting of the systematic review has been performed according to the PRISMA statements (10) and the Cochrane Handbook for Systematic Reviews of Diagnostic Test Accuracy (DTA) (11). The search was conducted in July 2018 and the latest included study was published in May 2018.

### Inclusion and Exclusion Criteria

Peer-reviewed studies were included in the analysis if pCMR and/or DSE were used to identify relevant coronary artery stenosis in patients at the age of 18 years and above with non-diagnosed or stable, asymptomatic CAD without ischemia-associated ECG abnormalities (right/left bundle branch block) and preserved left-ventricular ejection fraction. Only studies that included CCA and/or FFR as the reference test have been selected and sufficient detail to reconstruct a contingency table [e.g., true positive (TP), false positive (FP), false negative (FN), and true negative (TN) findings] was also needed. For pCMR, studies needed to use either adenosine or regadenoson perfusion with a qualitative or semi-quantitative approach and reports of CMR with dobutamine were not included. For DSE only studies with transthoracic assessment evaluating wall motion abnormalities were included.

Studies on animals, studies with fewer than 20 patients, and studies reporting data on patients with unstable angina, acute or subacute MI, heart transplantation, acute coronary revascularization, congenital or ischemic heart disease were excluded. Any studies using only physical stress, echo perfusion imaging or non-visual assessment were excluded. Studies with a different definition of relevant CAD determined by CCA and FFR were excluded, such as grade of stenosis <70% or a value >0.80 on FFR recordings (2). Studies on microvascular disease were also excluded. Studies in a language other than English, French or German were excluded.

### Search Methods for Identification of Studies

For the systematic review, MEDLINE (1946 to July 29, 2018), EMBASE (1974 to July 29, 2018) and Cochrane Library (Cochrane Database of Systematic Reviews: Issue 7 of 12, July 2018) databases were searched for articles that met inclusion criteria. Additionally, references of other meta-analyses published on the topic have been screened for further studies.

We developed a sensitive search strategy for MEDLINE (Ovid Web), EMBASE (Ovid Web) and the Cochrane Library (Wiley Online Library) as recommended in the Cochrane Handbook for Systematic reviews of DTA. The search strategy is shown in full in the Supplementary Material.

### Data Collection and Analysis

#### Selection of Studies

Two investigators (SMH and SIH) independently reviewed first article titles, then abstracts and finally the full text for eligibility. Discrepancies were resolved by discussion and consequent consensus.

#### Data Extraction and Management

For each study, both investigators (SMH and SIH) independently extracted information on author, year of publication, imaging technique, study size, demographic characteristics of participants (mean age, percentage male), magnetic field strength, type of stressor, type of assessment (qualitative or quantitative), definition of relevant CAD, prevalence of CAD and the presence of risk factors (diabetes, hypertension), the clinical settings considered (suspected or known CAD), as well as the numbers of TP, FP, FN, and TN. Discrepancies between investigators extraction were resolved by consensus after discussion.

If studies reported data for multiple CAD definitions (for instance at >50, >70, and >90% stenosis), only the sensitivity of the cut-off point that was the closest to our definition (e.g., >70%) was extracted. If a study reported sensitivity and specificity measures of multiple observers, the mean values were used. Patient characteristics extracted from all studies included in this meta-analysis are weight-adjusted averages; the weights have been based on the study size.

#### Assessment of Methodological Quality

The *Quality Assessment of Diagnostic Accuracy Studies* (QUADAS) tool (12), recommended in the modified version suggested by the *Cochrane Handbook for Systematic reviews of DTA* (13) was used to assess the quality of included studies. Two investigators (SMH and SIH) independently examined the study quality of the included reports. Disagreement was resolved by discussion and subsequent consensus.

### Statistical Analysis and Data Synthesis

For all included studies sensitivity, specificity, positive likelihood ratio (LR), negative LR, and diagnostic odds ratio (DOR) with 95% confidence interval (CI) were calculated from the TP, FP, FN, and TN results. Hierarchical models, recommended by the *Cochrane DTA reviews* (14), include the interdependence of sensitivity and specificity observed across studies which may alter their true effect size. Since an explicit cut-off point for relevant coronary artery stenosis was pre-defined in this meta-analysis, the *Bivariate model* by Reitsma (13) has been applied to produce summary operating points of sensitivity and specificity directly.

Statistical analysis was performed with Stata software version 15.0 (StataCorp LLC, College Station, Texas, USA). A two-tailed *p*-value of < 0.05 was considered to be significant, unless otherwise stated.

#### Investigations of Heterogeneity

One major cause of heterogeneity in test accuracy studies is the threshold effect (9). Therefore, only studies with the same reference value of relevant coronary stenosis are incorporated in this meta-analysis. Regardless, a meta-regression analysis has been facilitated to study potential reasons of heterogeneity in form of sex, age, sample size, MRI field strength, demographic patient characteristics, prevalence of CAD and several cardiovascular risk factors, as well as the reference method (CCA vs. FFR).

In-between study heterogeneity has been evaluated using the Cochrane-Q test (with a *p*-value of < 0.10 contemplating significant heterogeneity) and the *I*^2^ statistic (15).

#### Sensitivity Analysis

Pooled estimates of sensitivity and specificity with a 95% CI have been combined independently across all studies using a random effect meta-analysis that takes into account the possibility that these estimates may actually differ in-between studies, as a result of clinical and methodological differences (15). The value of LRs allows to compute the post-test probability based on Bayes' theorem (16).

#### Assessment of Reporting Bias

To explore publication bias, a funnel plot of the natural logarithm of the diagnostic odds ratio was constructed and a regression test for asymmetry was performed weighted to the study size (17). The threshold of significance was set to a *p*-value < 0.10 for this method.

## Results

### Literature Search

The systemic search identified 5,634 potentially relevant articles. After removal of duplicates and screening study titles, 1,306 articles were retained. These articles were screened by abstract and after 441 articles were excluded, the full texts of 865 articles were reviewed. Of these, forty-seven studies were judged as eligible for the meta-analysis.

**Figure 1:**
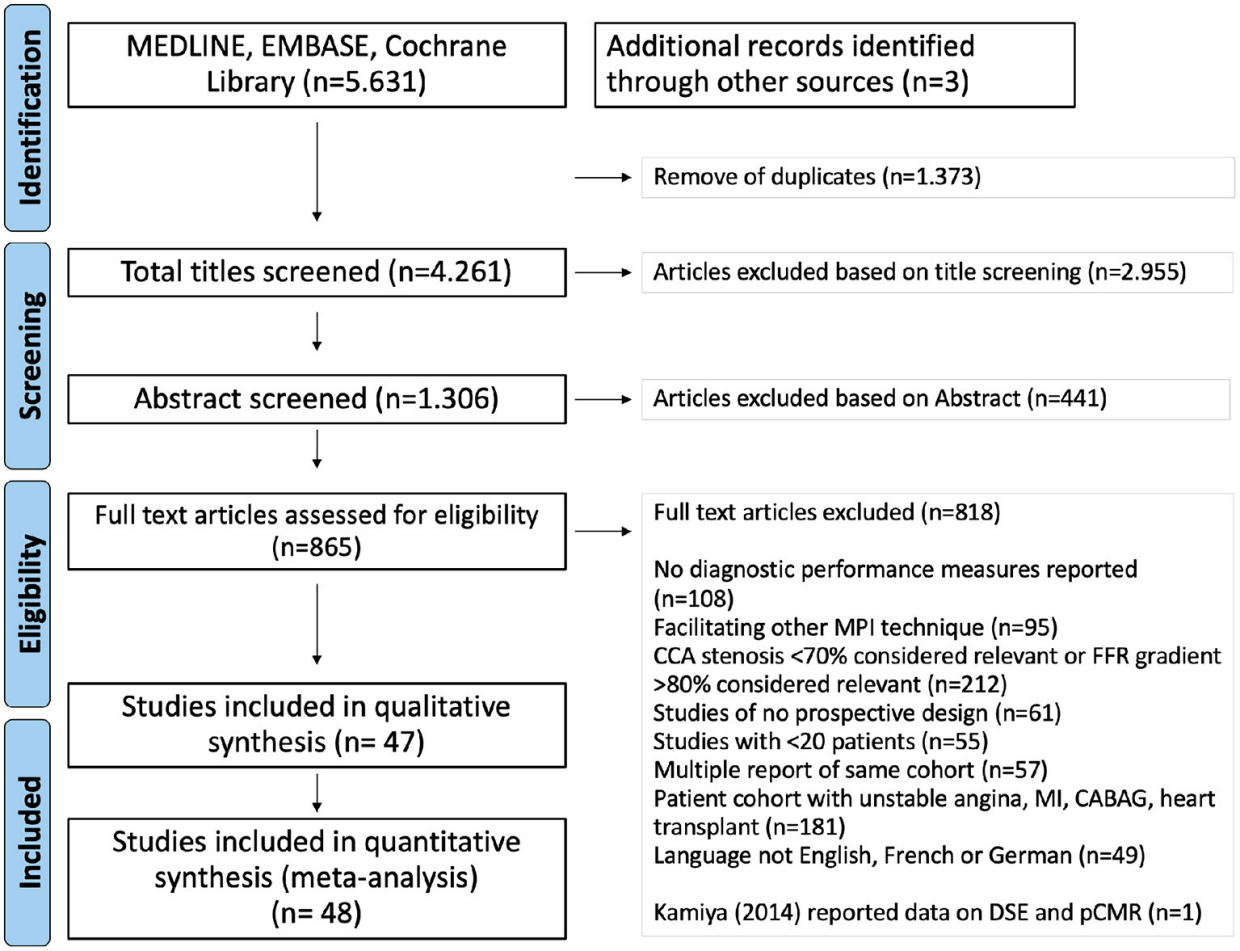
Flowchart of study selection process. Kamiya et al. (18) reported results for DSE and pCMR, so that it contributed with two sets of data to the quantitative, but only once to the qualitative assessment.

The flowchart of the article search and selection process is illustrated in Figure 1.

### Methodological Quality of Included Studies

Quality assessments using the QUADAS tool assessment can be found in the Supplementary Figure A1.

### Study Characteristics

A total of 47 studies with 4,742 patients, published between 1993 and 2018, were included in this meta-analysis: 39 pCMR (4,115 patients), 9 DSE (652 patients). One of these 47 studies, Kamiya et al. (18) reported both pCMR and DSE results, thus both sets of data contribed to the quantitative assessment. The systematic literature search initially identified more DSE studies (3,752 vs. 2,174) on the topic, however, the rigor of study design was generally inferior, so that only 0.2% as compared to 1.8% for pCMR studies fulfilled all of our strict inclusion criteria. The sample size varied from 24 to 676 patients. Results showed that 50% of patients (2,359 of 4,692) had hemodynamic relevant CAD (Table 1). The study populations had a mean age of 61 years and the majority of patients were men (64% of all patients). In most studies patients were hypertensive (60% of all patients) with some having additional risk factors, such as diabetes (21% of all patients).

**Table 1:** Study characteristics.

**Study**		**Imaging modality**	**MRI field strength**	**Study size**	**Data acquisition per**	**Reference test**	**CAD status**	**Mean age**	**Prevalence of CAD**	**Percentage male**	**Prevalence of diabetes**	**Prevalence of hypertension**
1	Hoffmann (1993)	DSE		64	Person	CCA	S	57	76%	77%	n/s	n/s
2	Dagianti (1995)	DSE		64	Person	CCA	S	55	39%	70%	n/s	n/s
3	Sochowski (1995)	DSE		46	Person	CCA	S	58	52%	67%	n/s	n/s
4	Bartunek (1996)	DSE		75	Person	FFR	S&K	57	72%	89%	n/s	n/s
5	Santoro (1998)	DSE		60	Person	CCA	S	n/s	55%	52%	n/s	n/s
6	Rieber (2004)	DSE		46	Person	FFR	S&K	64	65%	60%	21%	69%
7	Jung (2008)	DSE		70	Person	FFR	S&K	65	41%	64%	20%	76%
8	Kamiya (2014)	DSE		25	Vessel	FFR	S&K	68	41%	56%	60%	64%
9	Kim (2016)	DSE		202	Person	CCA	S	58	21%	0%	17%	43%
10	Nagel (2003)	pCMR	1.5	84	Person	CCA	S	63	51%	81%	0%	0%
11	Paetsch (2004)	pCMR	1.5	79	Person	CCA	S&K	61	38%	66%	24%	78%
12	Pons Lladó (2004)	pCMR	1.5	32	Vessel	CCA	S	65	72%	81%	31%	53%
13	Wolff (2004)	pCMR	1.5	75	Person	CCA	S&K	57	62%	83%	n/s	n/s
14	Plein (2005)	pCMR	1.5	92	Person	CCA	S&K	58	64%	74%	9%	33%
15	Klem (2006)	pCMR	1.5	92	Person	CCA	S	58	40%	49%	23%	64%
16	Pilz (2006)	pCMR	1.5	171	Person	CCA	S&K	62	66%	63%	27%	61%
17	Costa (2007)	pCMR	1.5	30	Vessel	FFR	n/s	65	47%	53%	23%	80%
18	Kühl (2007)	pCMR	1.5	28	Vessel	FFR	S&K	63	68%	61%	25%	64%
19	Merkle (2007)	pCMR	1.5	228	Person	CCA	S&K	61	67%	79%	20%	69%
20	Klem (2008)	pCMR	1.5	136	Person	CCA	S	63	27%	0%	22%	68%
21	Meyer (2008)	pCMR	3.0	60	Person	CCA	S	59	60%	63%	23%	65%
22	Watkins (2009)	pCMR	1.5	101	Person	FFR	S	60	77%	74%	16%	62%
23	Klumpp (2010)	pCMR	3.0	57	Vessel	CCA	S&K	62	72%	82%	25%	68%
24	Scheffel (2010)	pCMR	1.5	43	Vessel	CCA	S	64	65%	79%	19%	72%
25	Kirschbaum (2011)	pCMR	1.5	50	Vessel	FFR	S	64	n/s	76%	18%	50%
26	Lockie (2011)	pCMR	3.0	42	Vessel	FFR	S&K	57	52%	79%	19%	48%
27	Huber (2012)	pCMR	1.5	31	Vessel	FFR	S	67	55%	87%	23%	35%
28	Jogiya (2012)	pCMR	3.0	53	Vessel	FFR	S&K	64	61%	77%	30%	66%
29	Khoo (2012)	pCMR	1.5	241	Person	CCA	S&K	65	71%	n/s	n/s	n/s
30	Manka (2012)	pCMR	1.5	120	Person	FFR	S&K	64	58%	75%	26%	73%
31	Bernhardt (2013)	pCMR	3.0	34	Person	FFR	S	62	62%	76%	15%	79%
32	Bettencourt (2013)	pCMR	1.5	101	Person	FFR	S	62	44%	67%	39%	72%
33	Chiribiri (2013)	pCMR	3.0	67	Person	FFR	S&K	61	82%	79%	25%	48%
34	Ebersberger (2013)	pCMR	3.0	116	Person	FFR	S&K	63	78%	61%	26%	60%
35	Groothuis (2013)	pCMR	1.5	88	Person	FFR	S	56	30%	50%	12%	38%
36	Pereira (2013)	pCMR	1.5	80	Person	FFR	S	61	46%	68%	44%	72%
37	Walcher (2013)	pCMR	3.0	52	Vessel	CCA	S	62	52%	71%	19%	79%
8	Kamiya (2014)	pCMR	3.0	25	Vessel	FFR	S&K	68	41%	56%	60%	64%
38	Ponte (2014)	pCMR	1.5	95	Person	FFR	S	62	43%	68%	39%	75%
39	Greulich (2015)	pCMR	1.5	72	Person	CCA	S	70	14%	46%	28%	81%
40	Manka (2015)	pCMR	3.0	150	Person	FFR	S	63	57%	70%	18%	73%
41	Pan (2015)	pCMR	3.0	71	Vessel	FFR	S&K	60	57%	80%	31%	8%
42	Ripley (2015)	pCMR	1.5	676	Person	CCA	S	60	33%	62%	13%	51%
43	Papanastasiou (2016)	pCMR	3.0	24	Person	FFR	S&K	63	67%	83%	13%	54%
44	Foley (2017)	pCMR	1.5	54	Person	CCA	S&K	65	50%	53%	19%	54%
45	Hamada (2017)	pCMR	Mixed	357	Person	FFR	S&K	63	48%	85%	22%	76%
46	Biglands (2018)	pCMR	1.5	128	Person	CCA	S	61	33%	60%	13%	51%
47	Hsu (2018)	pCMR	1.5	80	Person	CCA	S&K	58	44%	70%	20%	69%

*n/s, not stated; S, suspected; S&K, suspected & known*.

### Diagnostic Accuracy

**Figure 2:**
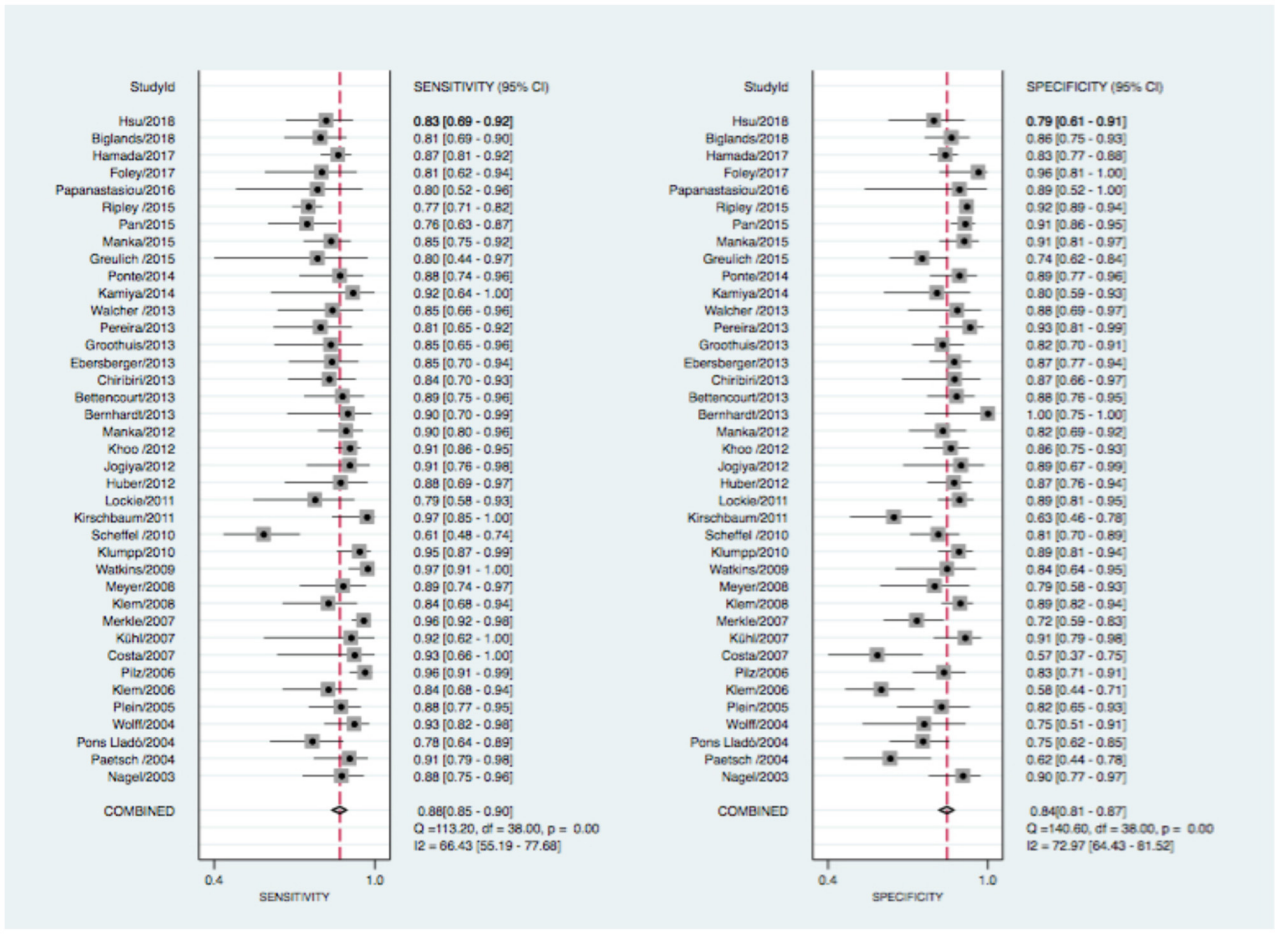
Forrest plot for pCMR with sensitivity (left) and specificity (right) estimates. CI, confidence intervall; I^2^, the percentage of variation across studies that is due to heterogeneity rather than chance; Q, Cochran's Q measure of heterogeneity.

**Figure 3:**
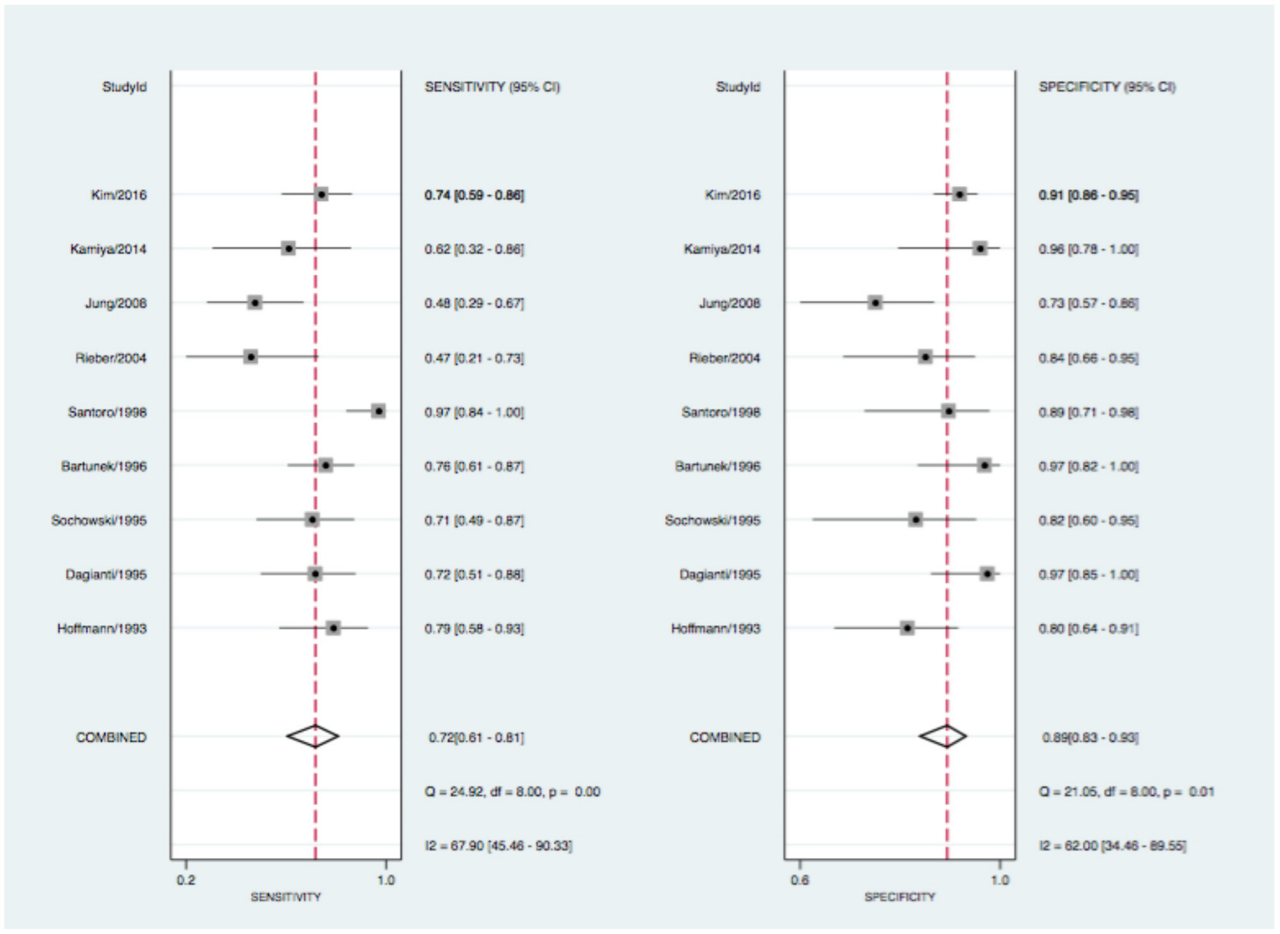
Forrest plot for DSE with sensitivity (left) and specificity (right) estimates. CI, confidence intervall; I^2^, the percentage of variation across studies that is due to heterogeneity rather than chance; Q, Cochran's Q measure of heterogeneity.

Pooled estimates of sensitivity, specificity, positive and negative LR, as well as DOR are summarized in Table 2. The data of all studies are summarized in forest plots (Figures 2, 3) and summary estimates of sensitivity and specificity for pCMR and DSE are stated with a 95% CI.

**Table 2:** Summary of findings table.

**Parameter**	***pCMR***		***DSE***	
	**Estimate**	**95% CI**	**Estimate**	**95% CI**
Number of studies included	**39**		**9**	
Number of patients included	**4.115**		**652**	
Sensitivity	**0.88**	[0.85–0.90]	**0.72**	[0.61–0.81]
Q	113.2	*p* < 0.1	24.9	*p* < 0.01
I^2^	66	[55–78]	68	[45–90]
Specificity	**0.84**	[0.81–0.87]	**0.89**	[0.83–0.93]
Q	140.6	*p* < 0.01	21.1	*p* < 0.01
I^2^	73	[64–82]	62	[34–90]
Positive LR	**5.5**	[4.7–6.5]	**6.3**	[3.8–10.4]
Negative LR	**0.14**	[0.12–0.18]	**0.31**	[0.21–0.46]
DOR	**38**	[29–49]	**20**	[9–46]
**Positive post-test probability**				
At 25% pre-test	65%		68%	
At 50% pre-test	85%		86%	
At 75% pre-test	94%		95%	
**Negative post-test probability**				
At 25% pre-test	5%		9%	
At 50% pre-test	13%		24%	
At 75% pre-test	30%		49%	
Deek's funnel plot *p*-value	**0.95**		**0.74**	

*DOR, diagnostic odds ratio; I^2^, the percentage of variation across studies that is due to heterogeneity rather than chance; LR, likelihood ratio; Q, Cochran's Q measure of heterogeneity*.

**Figure 4:**
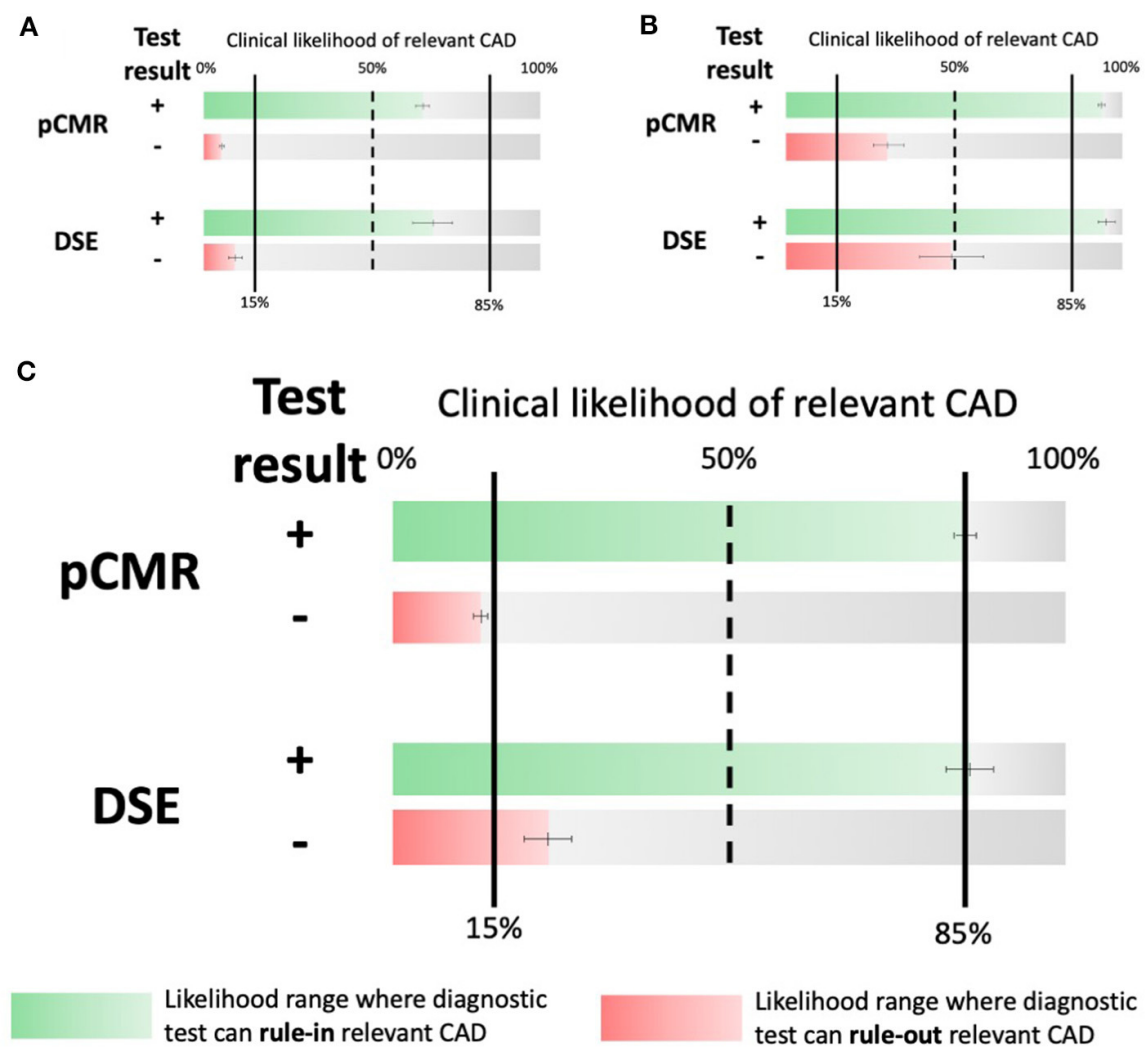
Central Illustration. Likelihood ranges at which the respective tests can rule-in (green) and rule-out (red) obstructive CAD. **(A)** Respective likelihood ranges at a low (25% PrTP). **(B)** a high risk (75% PrTP) of obstructive CAD and **(C)** Respective likelihood ranges at an intermediate risk of CAD (50% PrTP). Note that only pCMR can rule-out relevant CAD (defined as PoTP < 15%) at an intermediate PrTP. CAD, coronary artery disease; PrTP, pre-test probability; PoTP, post-test-probability.

At the patient level, pCMR (0.88, 95% CI: 0.85–0.90) had higher sensitivity compared to DSE (0.72, 95% CI: 0.61–0.81). Conversely, specificity of DSE (0.89, 95% CI: 0.83–0.93) was statistically non-superior compared to pCMR (0.84, 95% CI: 0.81–0.87), as described in Figures 2, 3. The DOR was highest for pCMR (38, 95% CI: 29–49) as compared to DSE (20, 95% CI: 9–46) (see Table 2). At a low clinical likelihood (pre-test probability 25%), both test fail to sufficiently rule-in (defined as post-test probability >85%) obstructive CAD (pCMR 65% CI: 63–67% vs. DSE 68% CI: 62–74%) (Figure 4A). In a patient with a very high likelihood (pre-test probability 75%) of CAD on the other hand, ruling out relevant stenosis (defined as post-test probability < 15%) becomes challenging when post-test probability ranges from CI: 26–35% for pCMR and CI: 39–58% for DSE studies (Figure 4B). In the intermediate risk cohort, however, with a pre-test probability of 50%, solemly an pCMR-based assessment could sufficiently rule-in and rule-out obstructive CAD with a post-test probability of 85% (CI: 83–86%), respectivley 13% (CI: 12–14%), compared to DSE-based assessment with 86% (CI: 83–90%), and 24% (CI: 21–28%) (Figure 4C).

Hints for heterogeneity were found for the sensitivity of pCMR (Q = 113.2, *p* < 0.01; I^2^ = 66, 95% CI: 55–78), and for the specificity results across studies (Q = 140.6, *p* < 0.01; I^2^ = 73, 95% CI: 64–82). DSE also showed heterogeneity for sensitivity (Q = 24.9, *p* < 0.01; I^2^ = 68, 95% CI: 45–90) and for specificity (Q = 21.1, *p* < 0.01; I^2^ = 72, 95% CI: 34–90) (see Figures 2, 3). The forest plots further highlights three potential outliers for pCMR studies Klem (2006), Costa (2007), Scheffel (2010), and a single potential outlier, Santoro (1998), for DSE studies. A sensitivity analysis is shown in the Supplementary Figures A2, A3 in which the influence of these studies on the summary estimates is assessed.

### Heterogeneity Assessment

**Figure 5:**
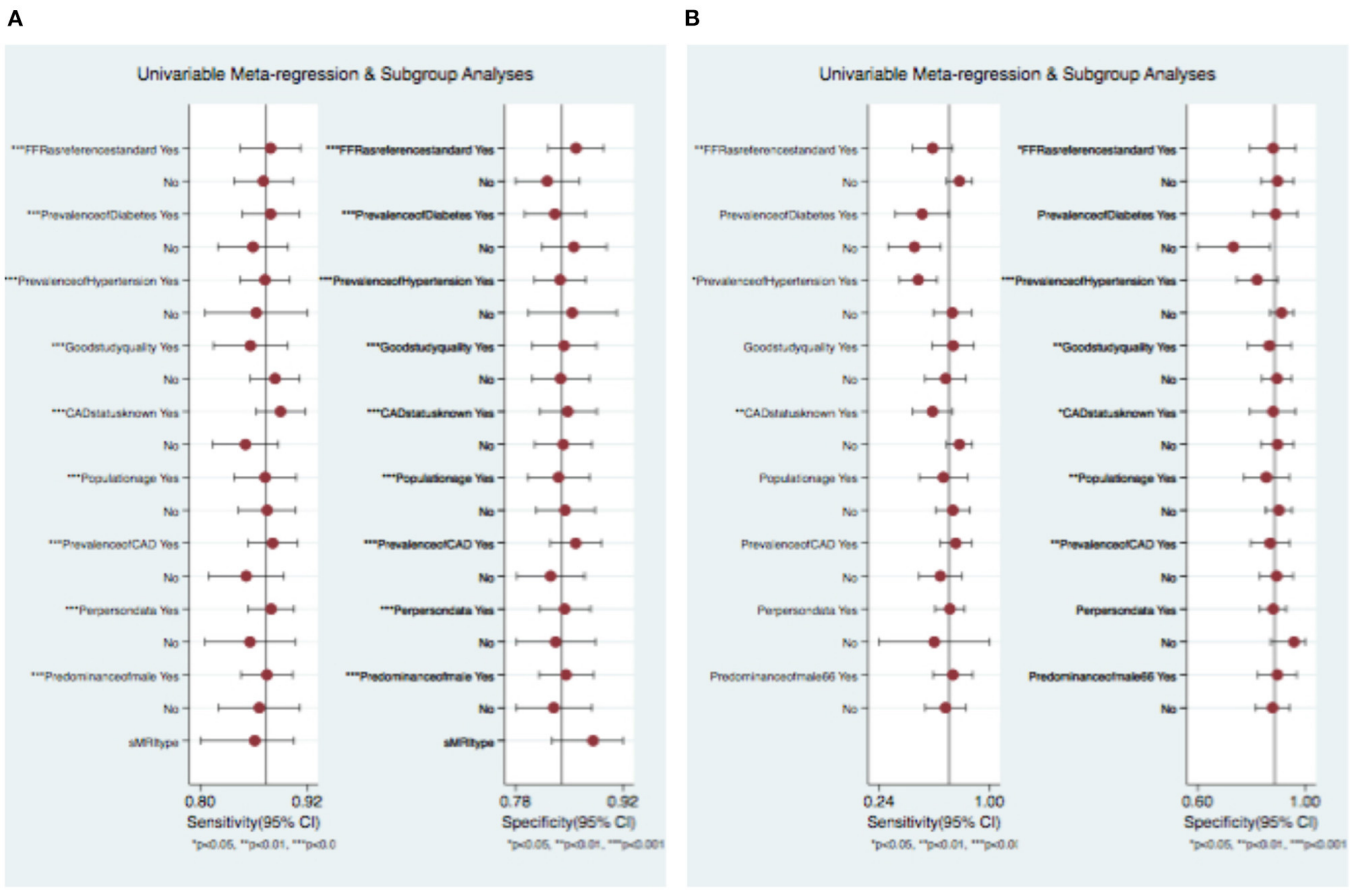
Univariable meta-regression and subgroup analysis of **(A)** pCMR and **(B)** DSE. CI, confidence intervall.

The meta-regression analysis was used in order to reveal factors impacting heterogeneity incorporated sex, age, MRI field strength, prevalence of CAD and cardiovascular risk factors, as well as the reference method (FFR vs. CCA). No parameter was identified as a significant predictor of heterogeneity for pCMR studies. For DSE studies, meta-regression analysis suggested that the prevalence of diabetes (*p* < 0.01) was an independent predictor of heterogeneity (see Figure 5).

In a subgroup analysis to identify reasons for in-between study variation and to investigate if the distribution of specific study characteristics biased the comparison of the two imaging methods, no significant effect of study and test characteristics was found on the DTA performance. A trend toward a lower diagnostic accuracy of pCMR studies performed at 1.5 T as compared to 3.0 T scanners was seen but was not significant. For DSE, studies with a higher prevalence of diabetes were associated with a worse diagnostic performance. The diagnostic accuracy of pCMR in comparison to DSE remained unaffected in the majority of subgroup analyses (see Figure 5). Surprisingly, the diagnostic accuracy of pCMR studies was not affected by the reference method, whereas DSE studies showed a tendency toward a lower sensitivity when using FFR rather than CCA as reference. This might hint an advantage of pCMR for the hemodynamic assessment of coronary stenosis over DSE, even though the differences did not reach a statistical significant level (*see* Figure 5).

A more in depth analysis of heterogeneity can be in the Supplementary Figure A4.

### Bias Assessment

**Figure 6:**
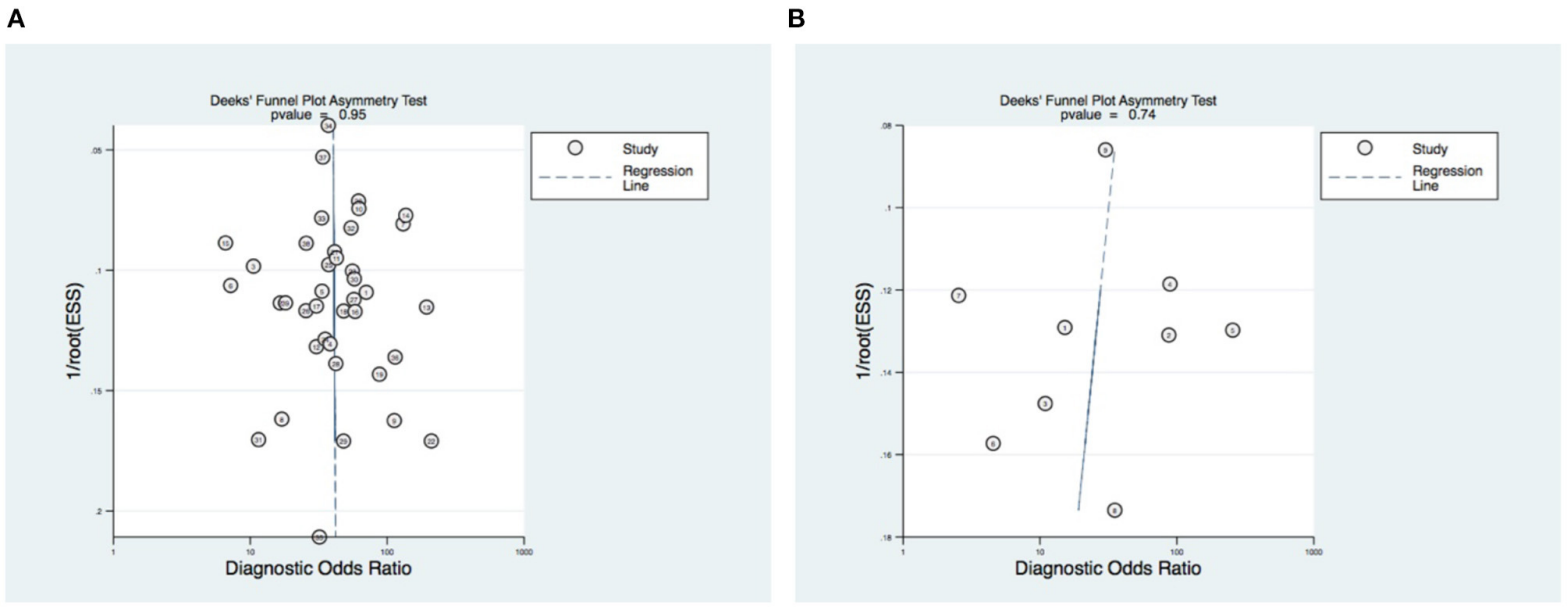
Publication bias assessment. Deek's funnel plot of **(A)** pCMR and **(B)** DSE. The vertical axis displays the inverse of the square root of the effective sample size [1/root(ESS)]. The horizontal axis displays the DOR. *P*-values <0.10 indicate non-symmetrical funnel shapes and is suggestive of publication bias. The subimage description at the end is then obsolete (**(A)** pCMR. **(B)** DSE).

In the assessment of publication bias, the slope coefficient for pCMR suggests symmetry in the data and therefore a low likelihood of publication bias with a statistically non-significant *p*-value of 0.95 (see Supplementary Material, Figure 6A). However, the slope in the DSE Deek's funnel plot (see Figure 6B) is suggestive of a bias from small studies even if the *p*-value is not significant (0.74).

## Discussion

### Findings

The present meta-analysis demonstrates that pCMR has a significantly higher diagnostic accuracy in detecting obstructive CAD than DSE in stable patients with an intermediate risk of CAD. Here, only an assessment with pCMR can rigourusly rule-in and rule-out relevant stenosis. Therefore, this meta-analysis can inform clinical decision making regarding interventional coronary therapy in the intermediate risk cohort. Despite the presently high utilization of DSE for non-invasive assessment of CAD especially in European facilities, the evidence for that is supported by studies of inferior quality and study populations with divers risk stratification (19). From the 3,752 studies initially identified by the systematic literature review for DSE in this meta-analysis, only nine studies (0.2%) had a high quality study design and fulfilled all inclusion criteria. This is a small sample of the available evidence on DTA of one of the oldest stress tests used in clinical practice to detect obstructive CAD. However, the broad body of evidence regarding DTA of DSE is very diverse in the sense of blended with comorbidities and preconditions, that we excluded in our very precise selection process in order to perfectly depict patients with an intermediate risk of CAD (20). Most studies had varying reference standards, unclear cut-off values of relevant stenosis, limited contemporary data, fewer comparisons to FFR or did not recruit a homogenous patient collective (4, 5). Less strict inclusion criteria would have allowed a larger proportion of studies to be included in the assessment of DTA of DSE, leading to more heterogeneity in-between studies and thus an overall wider confidence of respective results. By focussing on DSE and excluding studies with dypiridamole and/or adenosine stress-echocardiography we choose the modality with the highest diagnostic accuracy over its competitors at the expanse of a broader base of studies to include in our meta-analysis. This results in less heterogeneity, a less diluted and thus more comparable precision of DTA. However, the novelty of this meta-analysis is the assessment of a well-defined and very precise cohort of patients with an intermediate pre-test probability of CAD. Inclusion of studies with patients of other risk cohorts would have biased the results and falsified the interpretation of DTA for patients at an intermediate risk of CAD. Consequentley, our results precisely depict the DTA of pCMR and DSE for patient at an intermediate pre-test probability of CAD and cannot automatically be generalized to patients at other risk levels. Providing higher quality evidence on DSE is critical for clinical decision making and these issues should be adressed in large scale comparative-effectiveness trials, in order to reassess diagnostic recommendations (19).

The recent results of the ISCHEMIA trial put the necessity of testing for obstructive CAD in patients with intermediate risk in question (21). Arguably, this trial rather highlights the strong medical need to identify the optimal diagnostic test modality in this risk cohort, rather than making it obsolete. Since the identification of patients, where the benefits from invasive procedures outweigh the risks is essential, the results of our meta-analysis can guide clinical decision making in patients with an intermediate risk of obstructive CAD. The reservation must be made, however, that the non-invasive assessment in the ISCEHMIA trial was facilitated by MDCT, which can be susceptible to overestimating stenosis in specific patients (22). The non-inferiority of a strictly conservative vs. an invasive management could also be explained in part by a suboptimal identification of obstructive CAD, underlining the relevance of the evidence collated in this work.

Whilst not all commonly employed perfusion tests, such as SPECT, PET, or FFR_CT_ were included in this analysis, previous meta-analyses have suggested SPECT (DOR 9.1, 15.3) is less accurate than DSE (DOR 9.5, 26.3) and pCMR (DOR 92.2, 26.4) for the detection of relevant coronary stenosis (4, 5). However, more recent results from the EVINCI study, where a comparison between wall motion and myocardial perfusion imaging was performed in patients with stable chest pain and intermediate likelihood of CAD, demonstrate that the diagnostic accuracy of the latter was similar to that of DSE (23). It should be noted that perfusion imaging is predominately performed by SPECT in the USA, comprising around 90% of stress tests anually (24).

However, our findings cannot be generalized to other scenarios with patients at different pre-test probabilities or compared with myocardial perfusion tests not inlcuded in this assessment. Symptomatic patients with unstable disease or a high risk of CAD were excluded in this analysis because guidelines for this group are clear and supported by a large body of existing evidence (2, 3). Despite, the recently published results of the MR-INFORM trial provide evidence of non-inferiority of pCMR as compared to FFR in the symptomatic patient cohort with high risk of CAD, which might even influence future guideline recommendations in favor of pCMR (8). Nevertheless, current guidelines recommand non-invasive assessment strategies with MDCT in patients at low risk of obstructive CAD, since in this cohort perfusion tests failed to sustainably rule-out obstructive disease (2, 3).

In this meta-analysis, a non-inferior diagnostic accuracy of pCMR in the subgroup of diabetic patients could hint a relevant diagnostic advantage in patients with low event rates. On the contrary, DSE studies had an inferior diagnostic performance in this subgroup, which is inline with findings from the DIAD trial (25) and the work of van der Wall et al. (26), where the screening for CAD in asymptomatic, diabetic patients was ineffective given a low event rate. Nonetheless, current guidelines for symptomatic patients with a low risk for CAD are favoring MDCT for initial assessment, even though studies provide evidence of non-inferiority only to standard care and over a limited follow-up period of 6 months (3, 22). Results from this meta-analysis confirm a statistically insufficient risk startification in patients with low event rates with an non-invasive pCMR or DSE-based assessment. In consequence, these data emphasize the role of non-invasive imaging to guide clinical decision making and highlight the indisputable need for a distinct recommendation on the diagnostic work-up of patients with an intermediate risk of CAD.

### Strengths and Weaknesses of Review

The findings of this meta-analysis are novel and expand on previous studies (4, 5, 9). In contrast to previously published studies on DTA of obstructive CAD we like to emphasize that the results of our meta-analysis depict the clinically particularly relevant cohort of patients with an intermediate pre-test probability. Furthermore, we excluded studies that pre-selected their patients on the basis of angiographic findings to investigate the accuracy of the imaging modalities to identify obstructive CAD, rather than the ability to verify angiographic findings. This distinguishes our approach from the findings of the PACIFIC study for instance (27). Since the scope of our study was a patient- rather than a vessel-based assessment of DTA, our findings can be regarded as complementary to the results of the EVINCI trial, focusing on co-localization of perfusion defects with angiographic findings in patients also with an intermediate risk of CAD (23). In addition, a pre-defined, invasive cut-off value for diagnosis of relevant coronary artery stenosis has been applied so that we could compare more similar populations with less heterogenous results. This differentiates our work from previous meta-analysis. Specifically, FFR as well as CCA were used as reference standards with their respective cut-offs from international guidelines (2, 3, 28).

CCA cannot always provide sufficient information on the hemodynamic significance of a coronary artery stenosis, as the landmark trials FAME (29) as well as FAMOUS-NSTEMI (30) have shown. Nevertheless, “*diffuse coronary atherosclerosis without focal stenosis at angiography*” (31) can cause a continuous pressure fall along the vessel length, “*due to increased rest-perfusion, hence a lower flow-reserve*” (31) and this can lead to FFR values below the ischemic threshold, even in the absence of relevant CAD (31). Due to these issues, the analysis in this study was not restricted to reports that performed only FFR with the aim of avoiding confounding related to a narrow endpoint. In addition, studies that compared pCMR exclusively to FFR, sensitivity and specificity for the diagnosis of functionally relevant CAD has been significantly higher than in studies where it was compared with CCA only (32). This could be due to confounding of non-flow limiting stenosis in studies using CCA as the reference test with hemodynamically insignificant cut-off values. This meta-analysis incorporated only studies with severe definitions of significance for coronary artery stenosis (lumen narrowing of >70% in CCA) (2).

A key strength of this study is that a more comprehensive and detailed search strategy was used than in existing meta-analyses (4, 5, 9), meaning that the present study has a higher chance of identifying all available literature on the aimed risk cohort. For example, a similar published meta-analysis by Danad et al. reported data comparing several myocardial perfusion tests but did not use a systematic review of the literature nor a specific patient risk stratification (4). They included only three studies on DSE and four pCMR studies as compared to the nine DSE and 39 pCMR studies included in this review. This supports the argument that more comprehensive methods were able to identify a wider range of existing work (4). Another strength is that while prior syntheses have included studies that have been evaluated with reference tests that comprised FFR and CCA assessments at different cut-off points (5), this study reports at specific thresholds which minimizes risk of bias (14). On that note, studies that only used FFR measurements were largely single center and small trials. Patients were often pre-selected due to their angiographic findings, which may also improve sensitivity at the cost of specificity. These circumstances thus alter the generalizability of results and present another reason for incorporating both, CCA and FFR, reference standards as was done in this study. Even though a higher sensitivity but a lower specificity was seen in pCMR studies, which predominately used FFR measurements as the reference standard, the meta-regression and sub-group analysis of this report attested no confounding of preselection. To elaborate on that, reporting different cut-off values for FFR (e.g., 0.75 or 0.70) would have further limited the generalizability of results and potentially increase the risk of the threshold bias for a higher sensitivity at the expense of a lower specificity (9).

Finally, as with any meta-analysis, limitations to this method include heterogeneity in between studies and presence of publication bias. An in-depth discussion of limitations to this study can be found in the Supplementary Material.

## Conclusion

This systematic review and meta-analysis concludes that pCMR is superior to DSE in the diagnosis of relevant coronary artery stenosis in patients with an intermediate pre-test probability of CAD. Patients in this cohort might benefit from primary pCMR assessment for risk stratification and to guide further invasive procedures.

## Clinical Competencies

The evaluation of the accuracy of pCMR and DSE for diagnosis of significant coronary artery stenosis is relevant for the appropriate management and risk stratification of patients with suspected or stable CAD. Erroneous interpretations of hemodynamic relevance of stenosis can lead to clinically unnecessary revascularizations without any prognostic benefit to patients. Moreover, the underlying systematic review revealed a discrepancy between the absolute amount of evidence on DSE assessment for CAD and its significance regarding valide risk stratification and adaquate reference methodes, which limits their clinical value.

## Translational Outlook

Even though pCMR presented here as the superior non-invasive method, this meta-analysis does not qualify to comment on the general validity of its superiority in the assessment of CAD. A comparative cost-effectiveness analysis is needed, in order to assess efficiency. It may be possible, in certain patient groups, that pCMR has some diagnostic advantages over FFR but this was outside the scope of the current review.

## Data Availability Statement

The original contributions presented in the study are included in the article/Supplementary Material, further inquiries can be directed to the corresponding author/s.

## Author Contributions

SMH acquired the data (scanned studies for eligibility and extracted data), performed statistical analysis, conceived and designed the meta-analysis, conceptualized the key intellectual content, wrote, and drafted the manuscript. SIH scanned the studies for eligibility and extracted data to square results with SMH. GH and SP participated in the design and helped to coordination the workflow. FB, MK, GH, and SP took part in the editing of the manuscript. SP complemented also to the key intellectual content of this paper. All authors contributed to the article and approved the submitted version.

## Conflict of Interest

SP provides consultancy to, owns stock and has also received research support for unrelated research to this article from Circle Cardiovascular Imaging, Inc., Calgary, Alberta, Canada. The remaining authors declare that the research was conducted in the absence of any commercial or financial relationships that could be construed as a potential conflict of interest.

## Abbreviations

CAD: coronary artery disease
CCA: conventional coronary angiography
CI: confidence interval (CI)
DSE: dobutamine stress echocardiography
DTA: diagnostic test accuracy
DOR: diagnostic odds ratio
FFR: fractional flow reserve
FP: false positive
FN: false negative
LR: likelihood ratio
MDCT: multi-detector computer-tomography
pCMR: perfusion cardiovascular magnetic resonance
HSROC: hierarchical summary receiver operating characteristic
SPECT: single-photon emission computed tomography
MDCT: multidetector computed tomography
MI: myocardial infarction
TP: true positive
TN: true negative
QUADAS: Quality Assessment of Diagnostic Accuracy Studies tool.
