# Supplementary material for: Vasodilator Myocardial Perfusion Cardiac Magnetic Resonance Imaging Is Superior to Dobutamine Stress Echocardiography in the Detection of Relevant Coronary Artery Stenosis: A Systematic Review and Meta-Analysis on Their Diagnostic Accuracy
Source: Front Cardiovasc Med. 2021 Mar 12;8:630846. doi: 10.3389/fcvm.2021.630846 (PMC7994268; doi:10.3389/fcvm.2021.630846)
Supplement: Supplementary file 1 [file Presentation_1.pdf]

Supplementary materials

Search strategy

Medline (Ovid)

#	Search term	Hits
1	exp Echocardiography/	123.503
2	echocardio*.tw	134.966
3	echo-cardio*.tw	309
4	1 or 2 or 3	178.142
5	exp dobutamine/	5.963
6	Vasodilator Agents/ or Exercise Test/ or "Severity of Illness Index"/ or Stress, Pharmalogical/ or Myocardial Perfusion Imaging/ or Pharmacologic Stress Testing.mp	316.330
7	Stress/ or Echocardiography, Stress/ or stress-echo.mp	2.904
8	5 or 6 or 7	322.058
9	4 and 8	18.455
10	exp Magnetic Resonance Imaging/	395.786
11	((magnetic Resonance or MR or NMR) adj3 (Imag* or tomograph* or scan*)).tw	258.908
12	(MRI or MRIs or NMRI or NMRIS).tw	207.802
13	10 or 11 or 12	527.215
14	exp Myocardial Perfusion Imaging/	3.591
15	exp Adenosine/	48.846
16	Magnetic Resonance Angiography/ or Contrast Media/ or Magnetic Resonance Imaging, Stress/ or stress-mr*.mp	97.577
17	adenos*.tw	119.593
18	regadenos*.tw	264
19	(stress* adj3 MR*).tw	1.924
20	6 or 14 or 15 or 16 or 17 or 18 or 19	550.622
21	13 and 20	61.316
22	exp Sensitivity/	529.160
23	exp Specificity/	529.160
24	(Sensitivity and Specificity).mp [mp=title, abstract, original title, name of substance word, subject heading word, floating sub-heading word, keyword heading word, protocol supplementary concept word, rare disease supplementary concept word, unique identifier, synonyms]	450.001
25	22 or 23 or 24	632.370
26	Coronary Artery Disease/ or Coronary Disease/ or Coronary Disease/ or Myocardial Ischemia/	213.108
27	exp Coronary Angiography/	59.295
28	(PTA or PTCA).tw	14.305
29	((percutaneous coronary or coronary) adj6 (angiography or angioplasty or angio)).tw	47.386
30	((transluminal or trans-luminal) adj6 coronary).tw	7.524
31	angioplast*.tw	41.192
32	26 or 27 or 28 or 29 or 30 or 31	288.939
33	25 and 32	23.013
34	9 and 33	2.045
35	21 and 33	1.210
36	34 or 35	3.145

EMBASE (Ovid)

#	Search term	Hits
1	exp Echocardiography/	297.149
2	echocardio*.tw	225.253
3	echo-cardio*.tw	762
4	1 or 2 or 3	334.144
5	exp dobutamine/	23.015
6	Vasodilator Agents/ or Exercise Test/ or "Severity of Illness Index"/ or Stress, Pharmalogical/ or Myocardial Perfusion Imaging/ or Pharmacologic Stress Testing.mp	101.339
7	Stress/ or Echocardiography, Stress/ or stress-echo.mp	140.126
8	5 or 6 or 7	255.574
9	4 and 8	22.812
10	exp Magnetic Resonance Imaging/	823.611
11	((magnetic Resonance or MR or NMR) adj3 (Imag* or tomograph* or scan*)).tw	327.281
12	(MRI or MRIs or NMRI or NMRIS).tw	355.343
13	10 or 11 or 12	887.169
14	exp Myocardial Perfusion Imaging/	7.670
15	exp Adenosine/	39.640
16	Magnetic Resonance Angiography/ or Contrast Media/ or Magnetic Resonance Imaging, Stress/ or stress-mr*.mp	86.098
17	adenos*.tw	138.547
18	regadenos*.tw	738
19	(stress* adj3 MR*).tw	2.705
20	6 or 14 or 15 or 16 or 17 or 18 or 19	334.784
21	13 and 20	54.509
22	exp Sensitivity/	0
23	exp Specificity/	0
24	(Sensitivity and Specificity).mp [mp=title, abstract, heading word, drug trade name, original title, device manufacturer, drug manufacturer, device trade name, keyword, floating subheading word, candidate term word]	435.546
25	22 or 23 or 24	435.546
26	Coronary Artery Disease/ or Coronary Disease/ or Coronary Disease/ or Myocardial Ischemia/	216.654
27	exp Coronary Angiography/	13.638
28	(PTA or PTCA).tw	19.835
29	((percutaneous coronary or coronary) adj6 (angiography or angioplasty or angio)).tw	72.442
30	((transluminal or trans-luminal) adj6 coronary).tw	8.692
31	angioplast*.tw	56.730
32	26 or 27 or 28 or 29 or 30 or 31	312.896
33	25 and 32	12.242
34	9 and 33	1.473
35	21 and 33	833
36	34 or 35	2.135

Cochrane Library (Wiley Online)

#	Search term	Hits
#1	MeSH descriptor: [Echocardiography] explode all trees	4.234
#2	echocardio*:ti,ab,kw (Word variations have been searched)	10.809
#3	echo-cardio*:ti,ab,kw (Word variations have been searched)	27
#4	#1 or #2 or #3	10.822
#5	MeSH descriptor: [Dobutamine] explode all trees	531
#6	Vasodilator Agents or Exercise Test or Severity of Illness Index or Stress, Pharmalogical or Myocardial Perfusion Imaging:ti,ab,kw (Word variations have been searched)	53.042
#7	MeSH descriptor: [Exercise Test] explode all trees	8.075
#8	Stress or Echocardiography, Stress or stress-echo:ti,ab,kw (Word variations have been searched)	40.847
#9	#5 or #6 or #7 or #8	89.889
#10	#4 and #9	2.648
#11	MeSH descriptor: [Magnetic Resonance Imaging] explode all trees	8.073
#12	((magnetic Resonance or MR or NMR) near/3 (Imag* or tomograph* or scan*)):ti,ab,kw (Word variations have been searched)	19.963
#13	MRI or MRIs or NMRI or NMRIS:ti,ab,kw (Word variations have been searched)	11.744
#14	#11 or #12 or #13	22.504
#15	MeSH descriptor: [Myocardial Perfusion Imaging] explode all trees	185
#16	MeSH descriptor: [Adenosine] explode all trees	1.405
#17	Magnetic Resonance Angiography or Contrast Media or Magnetic Resonance Imaging, Stress or stress-mr*:ti,ab,kw (Word variations have been searched)	6.685
#18	adenos*:ti,ab,kw (Word variations have been searched)	5.128
#19	regadenos*:ti,ab,kw (Word variations have been searched)	104
#20	stress* near/3 MR*:ti,ab,kw (Word variations have been searched)	52
#21	#6 or #15 or #16 or #17 or #18 or #19 or #20	64.202
#22	#14 and #21	3.545
#23	MeSH descriptor: [Sensitivity and Specificity] explode all trees	19.521
#24	Sensitivity:ti,ab,kw (Word variations have been searched)	50.913
#25	Specificity:ti,ab,kw (Word variations have been searched)	23.920
#26	#23 or #24 or #25	60.373
#27	MeSH descriptor: [Coronary Angiography] explode all trees	4.264
#28	Coronary Artery Disease or Coronar Disease or Coronary Disease or Myocardial Ischemia:ti,ab,kw (Word variations have been searched)	31.573
#29	((percutaneous coronary or coronary) near/6 (angiography or angioplasty or angio)):ti,ab,kw (Word variations have been searched)	10.607
#30	((transluminal or trans-luminal) near/6 coronary):ti,ab,kw (Word variations have been searched)	1.481
#31	angioplast*:ti,ab,kw (Word variations have been searched)	7.915
#32	(PTA or PTCA):ti,ab,kw (Word variations have been searched)	1.719
#33	#27 or #28 or #29 or #30 or #31 or #32	38.469
#34	#26 and #33	3.060
#35	#10 and #34	234
#36	#22 and #34	131
#37	#35 or #36	351

Review-specific tailoring of QUADAS

#	Topic	Description	Yes	unclear	No
1	Representative spectrum	*Was the spectrum of patients representative of the patients who will receive the test in practice?*	43	1	3
2	Acceptable reference standard	*Is the reference standard likely to classify the target condition correctly?*	47	0	0
3	Acceptable delay between tests	*Is the time period between reference standard and index test short enough to be reasonably sure that the target condition did not change between the two tests?*	33	10	4
4	Partial verification avoided	*Did the whole sample or a random selection of the sample, receive verification using the intended reference standard?*	38	4	5
5	Differential verification avoided	*Did patients receive the same reference standard irrespective of the index test result?*	46	0	1
6	Incorporation avoided	*Was the reference standard independent of the index test (i.e. the index test did not form part of the reference standard)?*	47	0	0
7	Index test results blinded	*Were the reference standard results interpreted without knowledge of the results of the index test?*	31	14	2
8	Reference standard results blinded	*Were the index test results interpreted without knowledge of the results of the reference standard?*	35	12	0
9	Relevant clinical information	*Were the same clinical data available when test results were interpreted as would be available when the test is used in practice?*	7	20	20
10	Uninterpretable results reported?	*Were uninterpretable/ intermediate test results reported?*	34	12	1
11	Withdrawals explained?	*Were withdrawals from the study explained?*	41	4	2

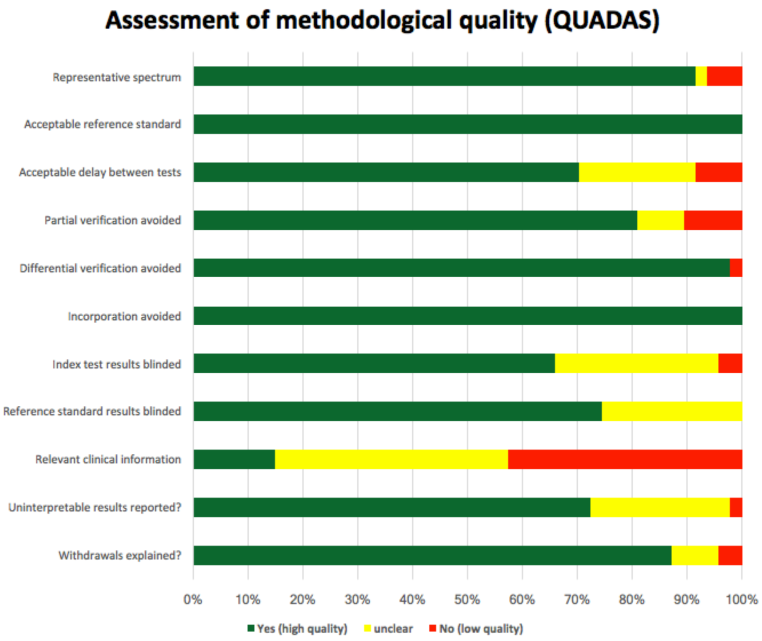

Supplementary material Figure A1: QUADAS tool assessment for all studies included in the meta-analysis.

Recommended quality items derived from QUADAS tool (Whiting 2003)

	Was the spectrum of patients representative of the patients who will receive the test in practice?<br>**Representative spectrum**<br>1	Is the reference standard likely to classify the target condition correctly?<br>**Acceptable reference standard**<br>2	Is the time period between reference standard and index test short enough to be reasonably sure that the target condition did not change between the two tests?<br>**Acceptable delay between tests**<br>3	Did the whole sample or a random selection of the sample, receive verification using the intended reference standard?<br>**Partial verification avoided**<br>4	Did patients receive the same reference standard irrespective of the index test result?<br>**Differential verification avoided**<br>5	Was the reference standard independent of the index test (i.e. the index test did not form part of the reference standard)?<br>**Incorporation avoided**<br>6	Were the reference standard results interpreted without knowledge of the results of the index test?<br>**Index test results blinded**<br>7	Were the index test results interpreted without knowledge of the results of the reference standard?<br>**Reference standard results blinded**<br>8	Were the same clinical data available when test results were interpreted as would be available when the test is used in practice?<br>**Relevant clinical information**<br>9	Were uninterpretable/ intermediate test results reported?<br>**Uninterpretable results reported?**<br>10	Were withdrawals from the study explained?<br>**Withdrawals explained?**<br>11
1 **Hoffmann (1993)**	good	good	good	unclear	good	good	good	good	bad	good	good
2 **Dagianti (1995)**	good	good	good	unclear	good	good	good	good	unclear	good	good
3 **Sochowski (1995)**	good	good	good	good	good	good	good	unclear	unclear	good	good
4 **Bartunek (1996)**	good	good	good	good	good	good	bad	good	bad	good	good
5 **Santoro (1998)**	good	good	good	good	good	good	unclear	good	good	good	good
6 **Rieber (2004)**	good	good	good	good	good	good	unclear	unclear	unclear	good	good
7 **Jung (2008)**	good	good	good	good	good	good	good	good	good	good	unclear
8 **Kamiya (2014)**	good	good	unclear	good	good	good	unclear	good	unclear	good	good
9 **Kim (2016)**	bad	good	good	bad	good	good	unclear	unclear	unclear	good	good
10 **Nagel (2003)**	bad	good	unclear	good	good	good	good	good	unclear	good	good
11 **Paetsch (2004)**	good	good	unclear	unclear	good	good	good	good	unclear	good	unclear
12 **Pons Lladó (2004)**	good	good	good	good	good	good	unclear	unclear	unclear	good	good
13 **Wolff (2004)**	good	good	good	good	good	good	good	good	unclear	good	good
14 **Plein (2005)**	good	good	good	good	good	good	good	good	bad	unclear	good
15 **Klem (2006)**	good	good	good	good	good	good	good	good	bad	good	good
16 **Pilz (2006)**	good	good	unclear	good	good	good	unclear	good	unclear	good	good
17 **Costa (2007)**	good	good	good	good	good	good	good	good	good	good	good
18 **Kühl (2007)**	good	good	good	good	good	good	good	good	good	good	good
19 **Merkle (2007)**	good	good	bad	good	good	good	good	good	bad	good	good
20 **Klem (2008)**	bad	good	good	good	good	good	good	good	bad	good	good
21 **Meyer (2008)**	good	good	good	good	good	good	unclear	good	unclear	good	bad
22 **Watkins (2009)**	good	good	unclear	good	good	good	good	good	bad	good	good
23 **Klumpp (2010)**	good	good	unclear	good	good	good	unclear	unclear	bad	good	bad
24 **Scheffel (2010)**	good	good	good	good	good	good	good	good	bad	unclear	good
25 **Kirschbaum (2011)**	good	good	good	good	bad	good	bad	unclear	good	unclear	unclear
26 **Lockie (2011)**	good	good	good	good	good	good	good	good	unclear	good	good
27 **Huber (2012)**	good	good	good	good	good	good	good	unclear	good	unclear	good
28 **Jogiya (2012)**	good	good	good	good	good	good	good	good	bad	good	good
29 **Khoo (2012)**	good	good	bad	bad	good	good	good	unclear	unclear	unclear	good
30 **Manka (2012)**	good	good	good	good	good	good	unclear	good	good	unclear	good
31 **Bernhardt (2013)**	unclear	good	good	unclear	good	good	unclear	unclear	unclear	unclear	unclear
32 **Bettencourt (2013)**	good	good	good	good	good	good	unclear	good	unclear	good	good
33 **Chiribiri (2013)**	good	good	bad	good	good	good	unclear	good	bad	bad	good
34 **Ebersberger (2013)**	good	good	good	good	good	good	good	unclear	bad	good	good
35 **Groothuis (2013)**	good	good	unclear	good	good	good	good	good	unclear	good	good
36 **Pereira (2013)**	good	good	good	good	good	good	unclear	good	bad	unclear	good
37 **Walcher (2013)**	good	good	good	good	good	good	good	good	bad	good	good
38 **Ponte (2014)**	good	good	good	good	good	good	good	good	bad	unclear	good
39 **Greulich (2015)**	good	good	good	good	good	good	good	good	bad	good	good
40 **Manka (2015)**	good	good	good	good	good	good	good	good	bad	good	good
41 **Pan (2015)**	good	good	good	good	good	good	good	good	bad	good	good
42 **Ripley (2015)**	good	good	good	good	good	good	good	good	unclear	good	good
43 **Papanastasiou (2016)**	good	good	unclear	good	good	good	unclear	good	bad	unclear	good
44 **Foley (2017)**	good	good	unclear	bad	good	good	good	unclear	unclear	unclear	good
45 **Hamada (2017)**	good	good	unclear	bad	good	good	good	good	bad	good	good
46 **Biglands (2018)**	good	good	good	good	good	good	good	good	unclear	unclear	good
47 **Hsu (2018)**	good	good	bad	bad	good	good	good	unclear	unclear	good	good

	pCMR	*DSE*
**Parameter**	**Estimate**	**Estimate**
E(logitSe)	**1.9777**	**0.9521**
E(logitSp)	**1.6648**	**2.0472**
Var(logitSe)	**0.2785**	**0.3798**
Var(logitSp)	**0.2569**	**0.2476**
Corr(logits)	**-0.0033**	**0.0259**

Supplementary material Table 1: Covariance matrix of parameter estimates among pCMR and DSE studies. *E(logitSe)* summary estimate for logit(sensitivity). *E(logitSp)* summary estimate for logit(specificity) and *Corr(logits)* their covariance. *Var(logitSe)* variances of the random effects for logit(sensitivity), *Var(logitSp)* of logit(specificity).

Influence of outlier detection analysis

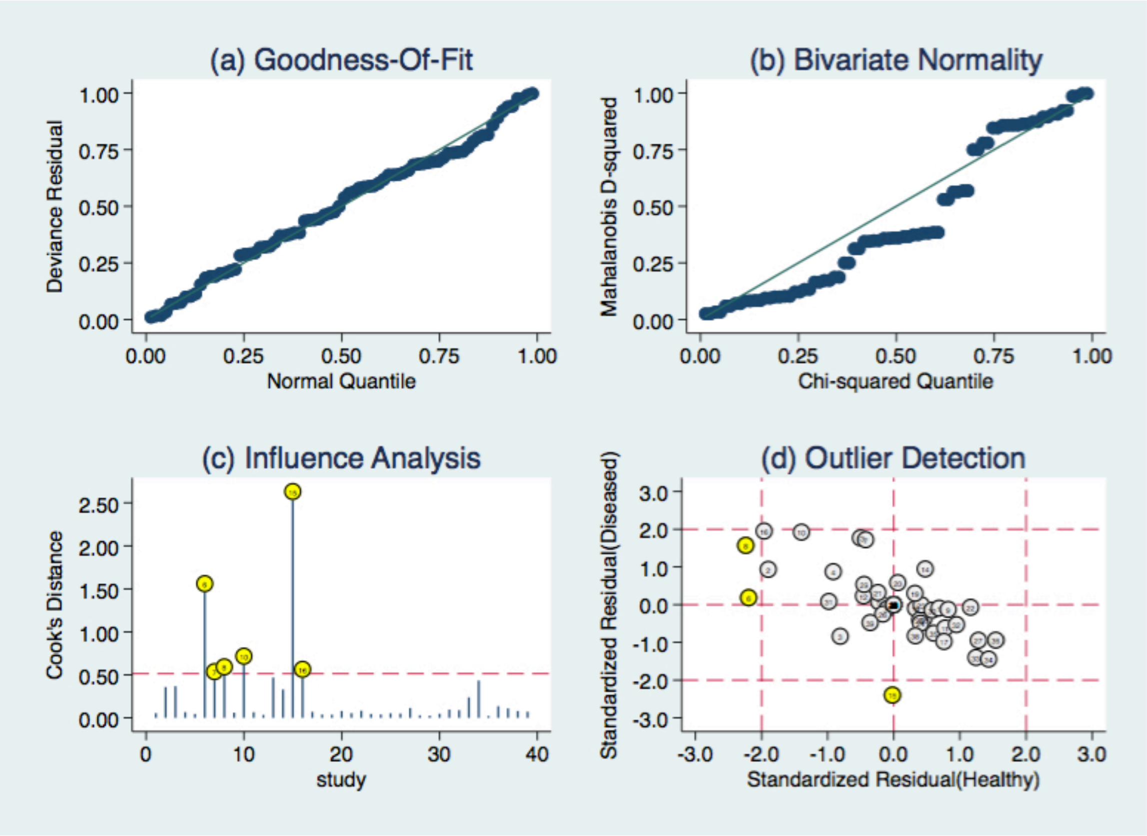

Supplementary material Figure A2: Influence of outlier analysis for sMRI studies with a) Goodness-of-Fit b) Bivariate Normality c) Influence analysis and d) Outlier detection.

A) Displays the Deviance residual on the y-axis and the normal quantile on the x-axis attesting an exceptional good fit of studies. B) Displays the Mahalano D-squared value on the y-axis and the Chi-squared quantile on the x-axis attesting normal distribution of sMRI studies. C) Displays the Cock's distance on the y-axis and the study number on the x-axis. Indicating a low influence of the three outliers (yellow) to the overall study results. D) Displays the standardized residual diseased on the y- and healthy on the x-axis with the three outliers in yellow and their relative position on the graph towards the overall study mean (black square in the graph centre).

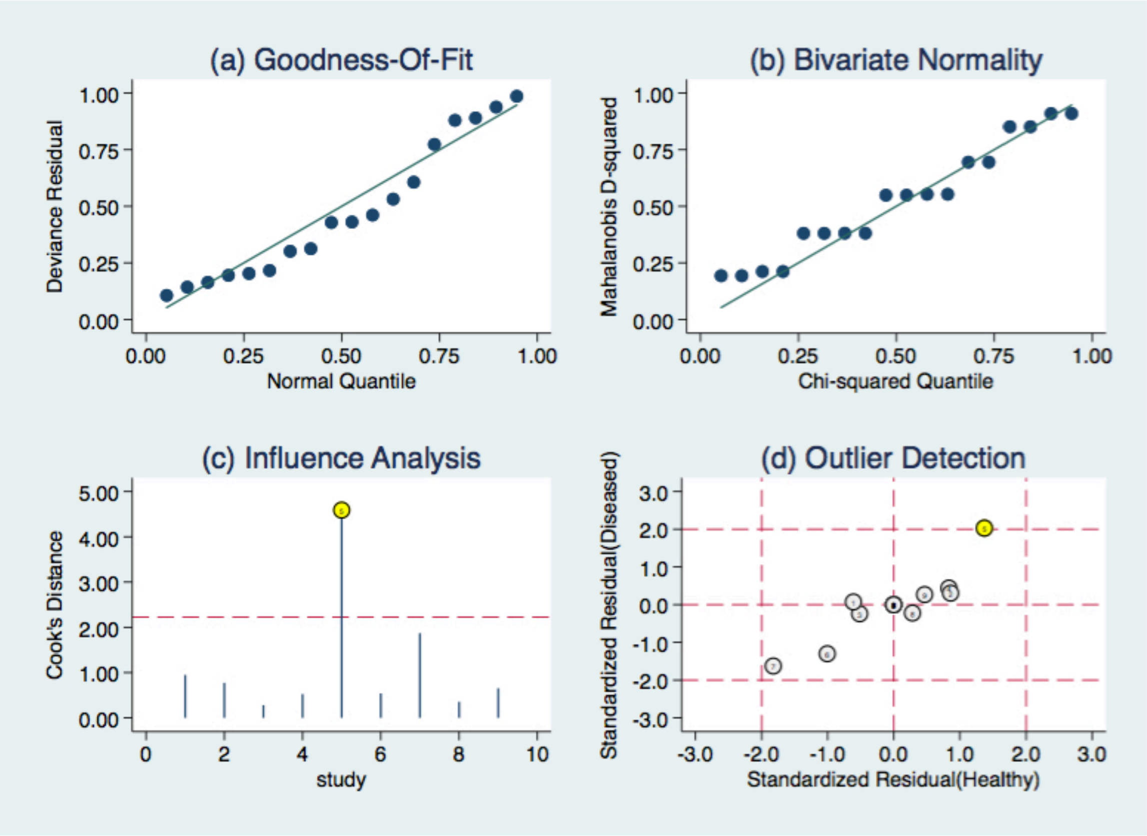

Supplementary material Figure A3: Influence of outlier analysis for DSE studies with a) Goodness-of-Fit b) Bivariate Normality c) Influence analysis and d) Outlier detection. A) Displays the Deviance residual on the y-axis and the normal quantile on the x-axis attesting an expectable good fit of studies. B) Displays the Mahalano D-squared value on the y-axis and the Chi-squared quantile on the x-axis attesting a roughly normal distribution of DSE studies. C) Displays the Cock's distance on the y-axis and the study number on the x-axis. Indicating a somewhat relevant influence of the outlier study (yellow) to the overall study results. D) Displays the standardized residual diseased on the y- and healthy on the x-axis with the outlier in yellow and its relative position on the graph towards the overall study mean (black square in the graph centre).

Moses-Littenberg SROC analysis

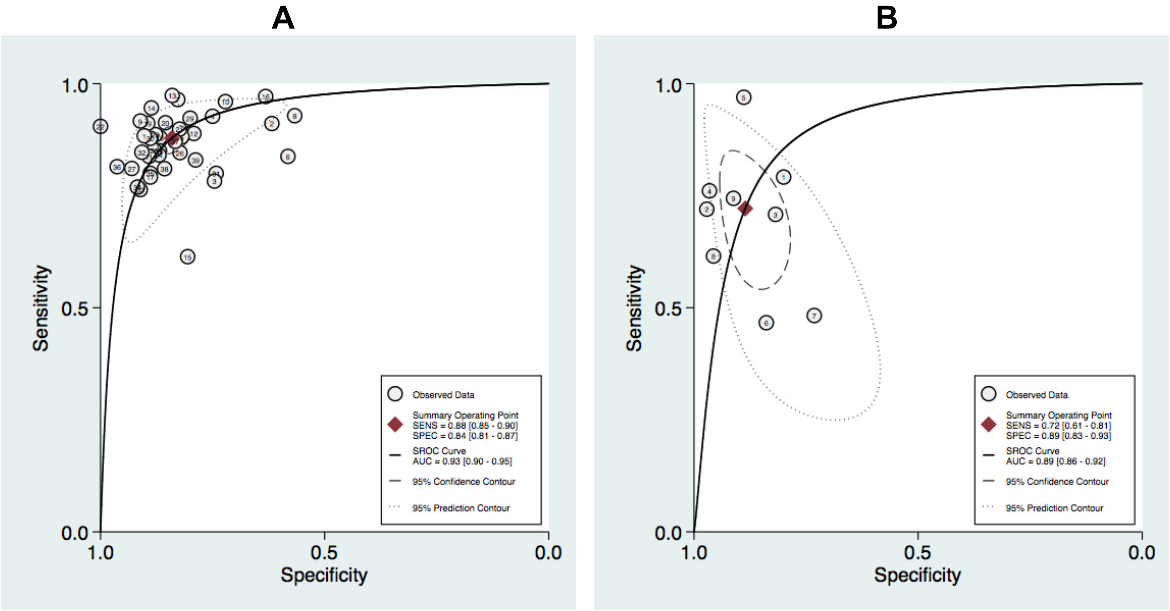

Supplementary material Figure A4: The vertical axis of the plots depicts sensitivity. The horizontal axis the specificity. The SROC curves (solid black) display the sensitivity as well as specificity of the individual studies with a 95% prediction (dotted line) and confidence (dashed line) region, complemented by the summary point in red for **A** sMRI and **B** DSE. The area under the SROC curve for sMRI is bigger as compared to the one of DSE (0.93 vs 0.89).

In depth analysis of across study variance

The outlier analysis depicted in *Supplementary material Figure A2* and *A3* implement an influence of one study (Klem 2008 - Cook's distance 2.7) to the pCMR results, and for one study (Santoro 1998 – Cook's distance 4.8) to the DSE results. The magnitude of heterogeneity can also be understood by the size of the prediction region in the SROC plot, (*Supplementary material Figure A4*) which attests pCMR studies a smaller degree of heterogeneity compared to DSE studies. The size of the prediction region on the SROC plot for pCMR (0.29; 95 % CI 0.11–0.77) was significantly smaller compared to DSE (1.07; 95 % CI 0.27–4.19; $p<0.01$). The sensitivity and specificity at this point is calculated by an "*inverse transformation of the logit estimates to give a sensitivity and specificity*"(1) of 0.88 and 0.84 in pCMR, and 0.72 and 0.89 in DSE studies, respectively. The significantly larger 95% confidence region in the SROC plot of DSE studies implies more uncertainty of the results and therefore a greater influence of the outlayer study.

Varying sample sizes of sensitivity and specificity measures in DSE studies as reasons for heterogeneity seem implausible since precision measures, such as the SROC plot give the impression of a broader heterogeneity of results.

In this meta-analysis only studies with a specifically defined threshold of significant coronary artery stenosis were included, which are then not randomly distributed accordingly. Nevertheless, it must be acknowledged that even a pre-defined cut-off value does not preclude some variance in the actual cut-off through differences in calibration, in the technical equipment, in the implementation, as well as between observers, causing additional heterogeneity of results.[22] It is therefore reasonable to estimate both, SORC and HSROC curves, as they may complement each other in the provision of clinically meaningful results.[22]

Study limitations

The subgroup analyses demonstrated most study characteristics did not significantly affect the diagnostic performance of pCMR and DSE. However, the power to detect relevant differences between subgroups may have been limited by the number of studies in specific subgroups, which tested DSE. The majority of studies included a mixture of patients with known and/or suspected CAD. Consequently, the subgroup analysis of CAD status (suspected or known) was limited due to unclear distinction of results in assorted cohorts. This meta-analysis suggests that the diagnostic accuracy of pCMR and DSE is not influenced by including patients with known CAD as far as

the data allow to comment.

Another limitation of meta-analyses in general is the dependence on the original publications in terms of quality and details of the reported data. Data on meaningful cardio-vascular risk factors, such as prevalence of smoking, hyperlipidaemia and family history, were generally poorly available and therefore not included in the analysis. Solely data on the prevalence of hypertension and diabetes were widely available. A more sophisticated cardiovascular risk assessment would have allowed to further assess heterogeneity among studies and could have increased in depth understanding of discrepancies in different diagnostic accuracy measures.

The heterogeneity across studies regarding the assessment methods, imaging techniques, setting, contrast agent, stressors used, patient inclusion criteria, CAD prevalence and percentage of male patients is a limitation of meta-analyses of DTAs. Although a specific CAD cut-off definitions and patient inclusion criteria were enabled, sample size limitations did not allow a subgroup analyses of all underlying factors. In this meta-analysis a random effect model was used, adjusting estimates and confidence intervals to the between-study variation. Nonetheless, the heterogeneity across studies remains a considerable limitation as stressed out above.

Additionally, one has to consider that the tested MPI examinations can also reveal functional information (e.g. left ventricular ejection fraction, presence of regional contraction abnormalities, presence of myocardial scars), more than an invasive assessment of coronary stenosis by CCA or a SPECT or PET image alone. The present analysis does not take the possible bias of these incidental parameters on the interpretation of the assessed MPI methods into account. Nevertheless, this information will also be present in the clinical evaluation of these MPIs and will therefore not increment their diagnostic accuracy. Noteworthy is also the fact that in the clinical routine at least a fraction of patients will be unsuitable for a sMRI assessment due to claustrophobia, BMI or severe renal failure. Like-wise, DSE can only be performed in presence of an adequate acoustic window, which limits the eligibility of some patients. Therefore, incompatible patients were excluded in the original papers, which in consequence, could influence the diagnostic accuracy of this meta-analysis. Sadly, there was no sufficient reporting of these information by the included studies to further elaborate this issue in a subgroup-analysis.

On a more in-depth review, did this meta-analysis incorporate results on per-patient as well as on per-vessel data. Thereby, the per-patient data is more valuable since we

treat individual patients and not individual coronaries. In theory, it is more plausible that the sensitivity of per-person level data is higher, however, the specificity is believed to be inferior. In practice however, the meta-regression analysis to this assessment could not confirm this theory and found the diagnostic accuracy in regards to per-person or –vessel data indifferent. Sensitivity 0.88 (95% CI: 0.86-0.91) vs 0.86 (95% CI: 0.81-0.91) and specificity 0.84 (95% CI: 0.81-0.88) vs 0.83 (95% CI: 0.78-0.88, $p < 0.01$).

The significant slope coefficient in the Deek's funnel plot (*Figure 6 b*) of DSE studies is suggestive of a significant small study bias. It is possible that small studies with a low diagnostic performance of DSE remained unpublished. Consequently, the presented diagnostic accuracy of DSE, which is still inferior to sMRI, could in fact be overestimated.

References of included studies

1. Hoffmann R, Lethen H, Kleinhans E, Weiss M, Flachskampf FA and Hanrath P. Comparative evaluation of bicycle and dobutamine stress echocardiography with perfusion scintigraphy and bicycle electrocardiogram for identification of coronary artery disease. *American Journal of Cardiology*. 1993;72:555-559.
2. Dagianti A, Penco M, Agati L, Sciomer S, Dagianti A, Rosanio S and Fedele F. Stress echocardiography: comparison of exercise, dipyridamole and dobutamine in detecting and predicting the extent of coronary artery disease.[Erratum appears in J Am Coll Cardiol 1995 Oct;26(4):1114]. *Journal of the American College of Cardiology*. 1995;26:18-25.
3. Sochowski RA, Yvorchuk KJ, Yang Y, Rattes MF and Chan KL. Dobutamine and dipyridamole stress echocardiography in patients with a low incidence of severe coronary artery disease. *Journal of the American Society of Echocardiography : official publication of the American Society of Echocardiography*. 1995;8:482-487.
4. Bartunek J, Marwick TH, Rodrigues AC, Vincent M, Van Schuerbeeck E, Sys SU and de Bruyne B. Dobutamine-induced wall motion abnormalities: correlations with myocardial fractional flow reserve and quantitative coronary angiography. *Journal of the American College of Cardiology*. 1996;27:1429-36.
5. Santoro GM, Sciagra R, Buonamici P, Consoli N, Mazzoni V, Zerauschek F, Bisi G and Fazzini PF. Head-to-head comparison of exercise stress testing, pharmacologic stress echocardiography, and perfusion tomography as first-line examination for chest pain in patients without history of coronary artery disease. *Journal of Nuclear Cardiology*. 1998;5:19-27.
6. Rieber J, Jung P, Erhard I, Koenig A, Hacker M, Schiele TM, Segmiller T, Stempfle HU, Theisen K, Siebert U and Klauss V. Comparison of pressure measurement, dobutamine contrast stress echocardiography and SPECT for the evaluation of intermerdiate coronary stenoses. The COMPRESS trial. *International Journal of Cardiovascular Interventions*. 2004;6:142-147.
7. Jung PH, Rieber J, Stork S, Hoyer C, Erhardt I, Nowotny A, Voelker W, Weidemann F, Ertl G, Klauss V and Angermann CE. Effect of contrast application on interpretability and diagnostic value of dobutamine stress echocardiography in patients with intermediate coronary lesions: comparison with myocardial fractional flow reserve. *European heart journal*. 2008;29:2536-43.
8. Kamiya K, Sakakibara M, Asakawa N, Yoshitani T, Iwano H, Komatsu H, Naya M, Chiba S, Yamada S, Manabe O, Kikuchi Y, Oyama-Manabe N, Oba K and Tsutsui H. Cardiac magnetic resonance performs better in the detection of functionally significant coronary artery Stenosis compared to single-photon emission computed Tomography and Dobutamine stress echocardiography. *Circulation Journal*. 2014;78:2468-2476.
9. Kim MN, Kim SA, Kim YH, Hong SJ, Park SM, Shin MS, Kim MA, Hong KS, Shin GJ and Shim WJ. Head to head comparison of stress echocardiography with exercise electrocardiography for the detection of coronary artery stenosis in women. *Journal of Cardiovascular Ultrasound*. 2016;24:135-143.
10. Nagel E, Klein C, Paetsch I, Hettwer S, Schnackenburg B, Wegscheider K and Fleck E. Magnetic resonance perfusion measurements for the noninvasive detection of coronary artery disease. *Circulation*. 2003;108:432-437.

11. Paetsch I, Jahnke C, Wahl A, Gebker R, Neuss M, Fleck E and Nagel E. Comparison of dobutamine stress magnetic resonance, adenosine stress magnetic resonance, and adenosine stress magnetic resonance perfusion. *Circulation*. 2004;110:835-842.

12. Pons Llado G, Carreras F, Leta R, Pujadas S and Garcia Picart J. Assesment of myocardial perfusion by cardiovascular magnetic resonance: Comparison with coronary angiography. [Spanish]. *Revista espanola de cardiologia*. 2004;57:388-395.

13. Wolff SD, Schwitter J, Coulden R, Friedrich MG, Bluemke DA, Biederman RW, Martin ET, Lansky AJ, Kashanian F, Foo TKF, Licato PE and Comeau CR. Myocardial first-pass perfusion magnetic resonance imaging: A multicenter dose-ranging study. *Circulation*. 2004;110:732-737.

14. Plein S, Radjenovic A, Ridgway JP, Barmby D, Greenwood JP, Ball SG and Sivananthan MU. Coronary artery disease: Myocardial perfusion MR imaging with sensitivity encoding versus conventional angiography. *Radiology*. 2005;235:423-430.

15. Klem I, Heitner JF, Shah DJ, Sketch Jr MH, Behar V, Weinsaft J, Cawley P, Parker M, Elliott M, Judd RM and Kim RJ. Improved Detection of Coronary Artery Disease by Stress Perfusion Cardiovascular Magnetic Resonance With the Use of Delayed Enhancement Infarction Imaging. *Journal of the American College of Cardiology*. 2006;47:1630-1638.

16. Pilz G, Bernhardt P, Klos M, Ali E, Wild M and Hofling B. Clinical implication of adenosine-stress cardiac magnetic resonance imaging as potential gatekeeper prior to invasive examination in patients with AHA/ACC class II indication for coronary angiography. *Clinical Research in Cardiology*. 2006;95:531-538.

17. Costa MA, Shoemaker S, Futamatsu H, Klassen C, Angiolillo DJ, Nguyen M, Siuciak A, Gilmore P, Zenni MM, Guzman L, Bass TA and Wilke N. Quantitative magnetic resonance perfusion imaging detects anatomic and physiologic coronary artery disease as measured by coronary angiography and fractional flow reserve. *Journal of the American College of Cardiology*. 2007;50:514-22.

18. Kuhl HP, Katoh M, Buhr C, Krombach GA, Hoffmann R, Rassaf T, Neizel M, Buecker A and Kelm M. Comparison of Magnetic Resonance Perfusion Imaging Versus Invasive Fractional Flow Reserve for Assessment of the Hemodynamic Significance of Epicardial Coronary Artery Stenosis. *American Journal of Cardiology*. 2007;99:1090-1095.

19. Merkle N, Wohrle J, Grebe O, Nusser T, Kunze M, Kestler HA, Kochs M and Hombach V. Assessment of myocardial perfusion for detection of coronary artery stenoses by steady-state, free-precession magnetic resonance first-pass imaging. *Heart*. 2007;93:1381-1385.

20. Klem I, Greulich S, Heitner JF, Kim H, Vogelsberg H, Kispert EM, Ambati SR, Bruch C, Parker M, Judd RM, Kim RJ and Sechtem U. Value of Cardiovascular Magnetic Resonance Stress Perfusion Testing for the Detection of Coronary Artery Disease in Women. *JACC: Cardiovascular Imaging*. 2008;1:436-445.

21. Meyer C, Strach K, Thomas D, Litt H, Nahle CP, Tiemann K, Schwenger U, Schild HH and Sommer T. High-resolution myocardial stress perfusion at 3 T in patients with suspected coronary artery disease. *European radiology*. 2008;18:226-233.

22. Watkins S, McGeoch R, Lyne J, Steedman T, Good R, McLaughlin MJ, Cunningham T, Bezlyak V, Ford I, Dargie HJ and Oldroyd KG. Validation of magnetic resonance myocardial perfusion imaging with fractional flow reserve for the detection of significant coronary heart disease. *Circulation*. 2009;120:2207-2213.

23. Klumpp BD, Seeger A, Doesch C, Doering J, Hoevelborn T, Kramer U, Fenchel M, Gawaz MP, Claussen CD and Miller S. High resolution myocardial magnetic resonance stress perfusion imaging at 3 T using a 1 M contrast agent. *European radiology*. 2010;20:533-541.

24. Scheffel H, Stolzmann P, Alkadhi H, Azemaj N, Plass A, Baumueller S, Desbiolles L, Leschka S, Kozerke S, Falk V, Boesiger P, Wyss C, Marincek B and Donati OF. Low-dose CT and cardiac MR for the diagnosis of coronary artery disease: Accuracy of single and combined approaches. *International Journal of Cardiovascular Imaging*. 2010;26:579-590.

25. Kirschbaum SW, Springeling T, Rossi A, Duckers E, Gutierrez-Chico JL, Regar E, De Feyter PJ and Van Geuns RJM. Comparison of adenosine magnetic resonance perfusion imaging with invasive coronary flow reserve and fractional flow reserve in patients with suspected coronary artery disease. *International Journal of Cardiology*. 2011;147:184-186.

26. Lockie T, Ishida M, Perera D, Chiribiri A, De Silva K, Kozerke S, Marber M, Nagel E, Rezavi R, Redwood S and Plein S. High-resolution magnetic resonance myocardial perfusion imaging at 3.0-Tesla to detect hemodynamically significant coronary stenoses as determined by fractional flow reserve. *Journal of the American College of Cardiology*. 2011;57:70-5.

27. Huber A, Sourbron S, Klauss V, Schaefer J, Bauner KU, Schweyer M, Reiser M, Rummeny E and Rieber J. Magnetic resonance perfusion of the myocardium: semiquantitative and quantitative evaluation in comparison with coronary angiography and fractional flow reserve. *Investigative Radiology*. 2012;47:332-8.

28. Jogiya R, Kozerke S, Morton G, De Silva K, Redwood S, Perera D, Nagel E and Plein S. Validation of dynamic 3-dimensional whole heart magnetic resonance myocardial perfusion imaging against fractional flow reserve for the detection of significant coronary artery disease. *Journal of the American College of Cardiology*. 2012;60:756-765.

29. Khoo JP, Grundy BJ, Steadman CD, Sonnex EP, Coulden RA and McCann GP. Stress cardiovascular MR in routine clinical practice: Referral patterns, accuracy, tolerance, safety and incidental findings. *British Journal of Radiology*. 2012;85:e851-e857.

30. Manka R, Paetsch I, Kozerke S, Moccetti M, Hoffmann R, Schroeder J, Reith S, Schnackenburg B, Gaemperli O, Wissmann L, Wyss CA, Kaufmann PA, Corti R, Boesiger P, Marx N, Luscher TF and Jahnke C. Whole-heart dynamic three-dimensional magnetic resonance perfusion imaging for the detection of coronary artery disease defined by fractional flow reserve: Determination of volumetric myocardial ischaemic burden and coronary lesion location. *European heart journal*. 2012;33:2016-2024.

31. Bernhardt P, Walcher T, Buckert D, Woehrle J and Rottbauer W. Adenosine blood oxygen level dependent T2-weighted imaging correlates to fractional flow reserve. *European heart journal*. 2013;1):1021.

32. Bettencourt N, Chiribiri A, Schuster A, Ferreira N, Sampaio F, Duarte R, Santos L, Melica B, Rodrigues A, Braga P, Teixeira M, Simoes L, Leite-Moreira A, Silva-Cardoso J, Nagel E, Portugal P and Gama V. Cardiac magnetic resonance myocardial perfusion imaging for detection of functionally significant obstructive coronary artery disease: A prospective study. *International Journal of Cardiology*. 2013;168:765-773.

33. Chiribiri A, Hautvast GLTF, Lockie T, Schuster A, Bigalke B, Olivotti L, Redwood SR, Breeuwer M, Plein S and Nagel E. Assessment of coronary artery stenosis severity and location: Quantitative analysis of transmural perfusion gradients by high-resolution MRI versus FFR. *JACC: Cardiovascular Imaging*. 2013;6:600-609.

34. Ebersberger U, Makowski MR, Schoepf UJ, Platz U, Schmidtler F, Rose J, Kessel A, Roth P, Antoni D, Schnackenburg B, Helmberger T, Rieber J, Hoffmann E and Leber AW. Magnetic resonance myocardial perfusion imaging at 3.0 Tesla for the identification of myocardial ischaemia: comparison with coronary catheter angiography and fractional flow reserve measurements. *European heart journal cardiovascular Imaging*. 2013;14:1174-80.

35. Groothuis JGJ, Beek AM, Brinckman SL, Meijerink MR, Van Den Oever MLP, Hofman MBM, Van Kuijk C and Van Rossum AC. Combined non-invasive functional and anatomical diagnostic work-up in clinical practice: The magnetic resonance and computed tomography in suspected coronary artery disease (MARCC) study. *European heart journal*. 2013;34:1990-1998.

36. Pereira E, Bettencourt N, Ferreira N, Schuster A, Chiribiri A, Primo J, Teixeira M, Simoes L, Leite-Moreira A, Silva-Cardoso J, Gama V and Nagel E. Incremental value of adenosine stress cardiac magnetic resonance in coronary artery disease detection. *International Journal of Cardiology*. 2013;168:4160-4167.

37. Walcher T, Ikuye K, Rottbauer W, Wohrle J and Bernhardt P. Is contrast-enhanced cardiac magnetic resonance imaging at 3 T superior to 1.5 T for detection of coronary artery disease? *International Journal of Cardiovascular Imaging*. 2013;29:355-361.

38. Ponte M, Bettencourt N, Pereira E, Ferreira ND, Chiribiri A, Schuster A, Albuquerque A, Gama V and Nagel E. Anatomical versus functional assessment of coronary artery disease: direct comparison of computed tomography coronary angiography and magnetic resonance myocardial perfusion imaging in patients with intermediate pre-test probability. *The international journal of cardiovascular imaging*. 2014;30:1589-97.

39. Greulich S, Steubing H, Birkmeier S, Grun S, Bentz K, Sechtem U and Mahrholdt H. Impact of arrhythmia on diagnostic performance of adenosine stress CMR in patients with suspected or known coronary artery disease. *Journal of Cardiovascular Magnetic Resonance*. 2015;17 (1) (no pagination).

40. Manka R, Gebker R, Wissmann L, Jogiya R, Motwani M, Frick M, Reinartz SD, Schnackenburg B, Nagel E, Plein S and Kozerke S. Multicenter evaluation of dynamic three-dimensional whole-heart myocardial perfusion imaging for the detection of coronary artery disease defined by fractional flow reserve. *Journal of Cardiovascular Magnetic Resonance*. 2013;1):190-191.

41. Pan J, Huang S, Lu Z, Li J, Wan Q, Zhang J, Gao C, Yang X and Wei M. Comparison of myocardial transmural perfusion gradient by magnetic resonance

imaging to fractional flow reserve in patients with suspected coronary artery disease. *American Journal of Cardiology*. 2015;115:1333-40.

42. Ripley DP, Motwani M, Brown JM, Nixon J, Everett CC, Bijsterveld P, Maredia N, Plein S and Greenwood JP. Individual component analysis of the multi-parametric cardiovascular magnetic resonance protocol in the CE-MARC trial. *Journal of Cardiovascular Magnetic Resonance*. 2015;17 (1) (no pagination).
43. Papanastasiou G, Williams MC, Dweck MR, Alam S, Cooper A, Mirsadraee S, Newby DE and Semple SI. Quantitative assessment of myocardial blood flow in coronary artery disease by cardiovascular magnetic resonance: Comparison of Fermi and distributed parameter modeling against invasive methods. *Journal of Cardiovascular Magnetic Resonance*. 2016;18 (1) (no pagination).
44. Foley JRJ, Kidambi A, Biglands JD, Maredia N, Dickinson CJ, Plein S and Greenwood JP. A comparison of cardiovascular magnetic resonance and single photon emission computed tomography (SPECT) perfusion imaging in left main stem or equivalent coronary artery disease: A CE-MARC substudy. *Journal of Cardiovascular Magnetic Resonance*. 2017;19 (1) (no pagination).
45. Hamada S, Gotschy A, Wissmann L, Paetsch I, Jahnke C, Plein S, Gebker R, Oebel S, Alkadhi H, Marx N, Luscher TF, Kozerke S and Manka R. Multi-centre study of whole-heart dynamic 3D cardiac magnetic resonance perfusion imaging for the detection of coronary artery disease defined by fractional flow reserve: Gender based analysis of diagnostic performance. *European Heart Journal Cardiovascular Imaging*. 2017;18:1099-1106.
46. Biglands JD, Ibraheem M, Magee DR, Radjenovic A, Plein S and Greenwood JP. Quantitative Myocardial Perfusion Imaging Versus Visual Analysis in Diagnosing Myocardial Ischemia: A CE-MARC Substudy. *JACC: Cardiovascular Imaging*. 2018;11:711-718.
47. Hsu LY, Jacobs M, Benovoy M, Ta AD, Conn HM, Winkler S, Greve AM, Chen MY, Shanbhag SM, Bandettini WP and Arai AE. Diagnostic Performance of Fully Automated Pixel-Wise Quantitative Myocardial Perfusion Imaging by Cardiovascular Magnetic Resonance. *JACC: Cardiovascular Imaging*. 2018;11:697-707.

Supplement material references

1. Reitsma JB, Rutjes AWS, Whiting P, Vlassov VV, Leeflang MMG, Deeks JJ. Chapter 9: Assessing methodological quality. , Version , 2009. Available from: : The Cochrane Collaboration (2009) [cited 2018 June 29th]. 1.0.0:[Available from: http://srdta.cochrane.org/.
